# Direct Clinical Applications of Natural Language Processing in Common Neurological Disorders: Scoping Review

**DOI:** 10.2196/51822

**Published:** 2024-05-22

**Authors:** Ilana Lefkovitz, Samantha Walsh, Leah J Blank, Nathalie Jetté, Benjamin R Kummer

**Affiliations:** 1 Department of Neurology Icahn School of Medicine at Mount Sinai New York, NY United States; 2 Hunter College Libraries Hunter College, City University of New York New York, NY United States; 3 Department of Population Health Science and Policy Icahn School of Medicine at Mount Sinai New York, NY United States; 4 Department of Clinical Neurosciences University of Calgary Calgary, AB Canada; 5 Clinical Neuro-Informatics Program Icahn School of Medicine at Mount Sinai New York, NY United States; 6 Windreich Department of Artificial Intelligence and Human Health Icahn School of Medicine at Mount Sinai New York, NY United States

**Keywords:** natural language processing, NLP, unstructured, text, machine learning, deep learning, neurology, headache disorders, migraine, Parkinson disease, cerebrovascular disease, stroke, transient ischemic attack, epilepsy, multiple sclerosis, cardiovascular, artificial intelligence, Parkinson, neurological, neurological disorder, scoping review, diagnosis, treatment, prediction

## Abstract

**Background:**

Natural language processing (NLP), a branch of artificial intelligence that analyzes unstructured language, is being increasingly used in health care. However, the extent to which NLP has been formally studied in neurological disorders remains unclear.

**Objective:**

We sought to characterize studies that applied NLP to the diagnosis, prediction, or treatment of common neurological disorders.

**Methods:**

This review followed the PRISMA-ScR (Preferred Reporting Items for Systematic Reviews and Meta-Analyses Extension for Scoping Reviews) standards. The search was conducted using MEDLINE and Embase on May 11, 2022. Studies of NLP use in migraine, Parkinson disease, Alzheimer disease, stroke and transient ischemic attack, epilepsy, or multiple sclerosis were included. We excluded conference abstracts, review papers, as well as studies involving heterogeneous clinical populations or indirect clinical uses of NLP. Study characteristics were extracted and analyzed using descriptive statistics. We did not aggregate measurements of performance in our review due to the high variability in study outcomes, which is the main limitation of the study.

**Results:**

In total, 916 studies were identified, of which 41 (4.5%) met all eligibility criteria and were included in the final review. Of the 41 included studies, the most frequently represented disorders were stroke and transient ischemic attack (n=20, 49%), followed by epilepsy (n=10, 24%), Alzheimer disease (n=6, 15%), and multiple sclerosis (n=5, 12%). We found no studies of NLP use in migraine or Parkinson disease that met our eligibility criteria. The main objective of NLP was diagnosis (n=20, 49%), followed by disease phenotyping (n=17, 41%), prognostication (n=9, 22%), and treatment (n=4, 10%). In total, 18 (44%) studies used only machine learning approaches, 6 (15%) used only rule-based methods, and 17 (41%) used both.

**Conclusions:**

We found that NLP was most commonly applied for diagnosis, implying a potential role for NLP in augmenting diagnostic accuracy in settings with limited access to neurological expertise. We also found several gaps in neurological NLP research, with few to no studies addressing certain disorders, which may suggest additional areas of inquiry.

**Trial Registration:**

Prospective Register of Systematic Reviews (PROSPERO) CRD42021228703; https://www.crd.york.ac.uk/PROSPERO/display_record.php?RecordID=228703

## Introduction

The implementation of the electronic medical record (EMR) in health care systems has resulted in a remarkable increase in the amount of digital patient data [1], much of which is text-based and stored in an unstructured, narrative format [2-4]. While unstructured text is a rich data source, analyses of these data often require time- and cost-intensive manual processing [3]. Natural language processing (NLP), a type of artificial intelligence that automatically derives meaning from unstructured language, can significantly reduce costs and enhance the quality of health care systems by converting unstructured text into a structured form that can be processed by computers [2,4,5].

Approaches to NLP can use rule-based techniques, machine learning (ML), or a combination of both [6-8]. Between the fifth and eighth decades of the 20th century, NLP approaches were predominantly rule-based, using a set of rules defined by human experts [7,9] to systematically extract meaning from unstructured text. Rule-based methods are comprehensible by humans but difficult to generalize [7,9]. Driven by recent advances in computing power and access to computing resources, contemporary approaches to NLP have increasingly incorporated ML, which possesses greater scalability [7] than rule-based methods despite the need for greater computational power to construct ML-based NLP models. Most recently, complex ML methods such as deep learning (DL), which are based on neural networks and larger datasets than conventional ML approaches, have become popular approaches to address NLP tasks [9,10].

The high prevalence of unstructured text in EMR systems creates an ideal use case for NLP in health care. However, the majority of current NLP research remains focused on nonneurological conditions such as mental health, cancer, and pneumonia [5]. The dearth of neurological NLP research is out of proportion to the worldwide importance of neurological conditions, both in terms of public health burden and cost. For instance, cerebrovascular disease occupies the second leading cause of death worldwide [11], and in the United States, neurological and musculoskeletal disorders generate the greatest number of years lost to disability [12]. Finally, the estimated annual cost of the most prevalent neurological diseases in the United States is nearly US $800 billion [12].

Neurology is a specialty that is uniquely well suited to benefit from NLP approaches. The data used in the diagnosis and management of neurological conditions, such as examination findings or clinical impressions, are often recorded as narrative, unstructured text in clinical documentation. Aside from clinical notes containing the patient history and neurological examination, reports from radiology [13,14], sonography, or electrophysiology studies are integral to neurological practice and often are crucial for detection, prognosis, and treatment. Further, NLP analysis of spoken language may allow the detection of certain neurodegenerative conditions such as Alzheimer disease in their early stages [15]. Given the unique position of neurology with respect to NLP and the relative lack of research on the applications of NLP in neurology, we sought to conduct a scoping review in order to quantify and characterize studies that directly applied NLP for clinical use in common neurological disorders.

## Methods

### Literature Search Strategy and Eligibility Criteria

This review was conducted using the PRISMA-ScR (Preferred Reporting Items for Systematic Reviews and Meta-Analyses Extension for Scoping Reviews) guidelines (Multimedia Appendix 1) and was registered with the Prospective Register of Systematic Reviews (PROSPERO CRD42021228703). Our search was conducted using Ovid Embase and MEDLINE on May 11, 2022 (Multimedia Appendix 2 [16-22]). Based on the most globally prevalent and costly neurological disorders [11], studies investigating the use of NLP in Alzheimer disease (exclusive of Alzheimer disease–related disorders), Parkinson disease, stroke and transient ischemic attack, epilepsy, multiple sclerosis (MS), and migraine were included.

Studies that used NLP to analyze radiographic findings without any clinical correlation (eg, silent brain infarcts) or for purposes other than diagnosis, detection, phenotyping, subtyping, prognostication, risk stratification, or therapy were excluded. We excluded studies with populations comprised of patients with heterogeneous diseases or ambiguously defined populations (eg, we excluded studies that used a patient cohort consisting of patients with both Alzheimer dementia and mild cognitive impairment) as well as studies that did not use NLP for direct clinical applications. Examples of indirect clinical applications include the use of NLP to identify cohorts for subsequent model development or conduct epidemiological associations between cohorts without direct impact on clinical practice. We additionally excluded abstracts, conference proceedings, reviews, and editorials.

### Data Extraction

A medical librarian (SW) with expertise in scoping reviews first conducted a literature search (Multimedia Appendix 2) based on our eligibility criteria to generate a list of abstracts, which were then imported into a web application (Covidence Ltd) for initial screening by 3 authors (BRK, LJB, and IL). After the abstract screening was completed, full-text papers for screened abstracts were reviewed by 2 authors (BRK and IL) to determine eligibility for inclusion. Disagreements at both stages were resolved by discussion and consensus.

Using the final list of full-text studies, study characteristics were manually extracted by 1 author (IL) and charted in a REDCap (Research Electronic Data Capture; REDCap Consortium) web database form, which was subsequently reviewed by a second author (BRK) for accuracy. The data charting form was initially tested by the data extractor (IL) and revised after feedback from all coauthors (BRK, NJ, LJB, and SW). We extracted study publication year, population size, country of origin, journal field (eg, medical informatics, clinical neurology, nonclinical neuroscience, clinical medicine, or other), neurological disorder, and target of NLP (eg, diagnosis or detection, phenotyping or subtyping and severity, prognostication or risk stratification, or disease management or therapy). Each study could have multiple targets whenever applicable.

For each study, the source language to which NLP techniques were applied was also extracted. For studies conducted in or authored by teams from non-English–speaking countries, the source language was extrapolated directly as described from the study methodology. If the source language was a publicly available research dataset or ontology (eg, MetaMap ontology or ADReSS dataset, both of which use English), the source language was reported as English. Source of language for NLP (eg, clinical notes, radiographic reports, speech audio, or other) and type of study (eg, model derivation, validation, or both) were also noted. Validation studies were defined as studies that specifically investigated the validation of a derived model in a population external to the original model derivation population. Our definition of validation studies did not include validation on held-out test sets as part of model derivation. If the NLP model was both derived and externally validated in the same study, the population size included the additional population used for validation. Simulated patients, who were used as a training set in one study, were included in the population size. If no population size was mentioned in the studies, the number of text instances (eg, clinical notes and radiographic reports) was recorded.

We additionally extracted the study’s NLP approaches (ie, rule-based methods, ML, or both). Rule-based NLP included any approaches that used keyword searches, pattern matching, regular expressions, or ontological systems for word-concept mapping, text preprocessing, or classification. ML-based NLP comprised both conventional ML and DL approaches and both were distinguished as dichotomous study characteristic variables but could co-occur in the studies. A study was characterized as including any of these methods if either ML or DL was used at any point in model development for the study.

Under the category of conventional ML methods, linear regression, logistic regression, support vector machines (SVMs), naïve Bayes classifiers, decision trees, random forest classifiers, k-nearest neighbor algorithms, gradient boosting techniques such as extreme gradient boosting, latent Dirichlet allocation, and shallow neural networks were included. Under the definition of shallow neural network, we included any approaches using Word2vec or other “-2vec” word-embedding techniques that use a neural network to construct word contexts and extract semantic and syntactic meaning from text [23,24]. We also included other types of regression, such as lasso regression, which is often used for dimensionality reduction, in the conventional ML category.

DL techniques included convolutional neural networks, recurrent neural networks (RNNs), long- and short-term memory networks, multilayer perceptrons, and transformers. Studies using long- and short-term memory networks were also categorized as using an RNN. We also note that neural networks of unspecified type and number of layers, which were not clearly referred to as DL in the study, were not included in this category.

## Results

### Included Studies

**Figure 1:**
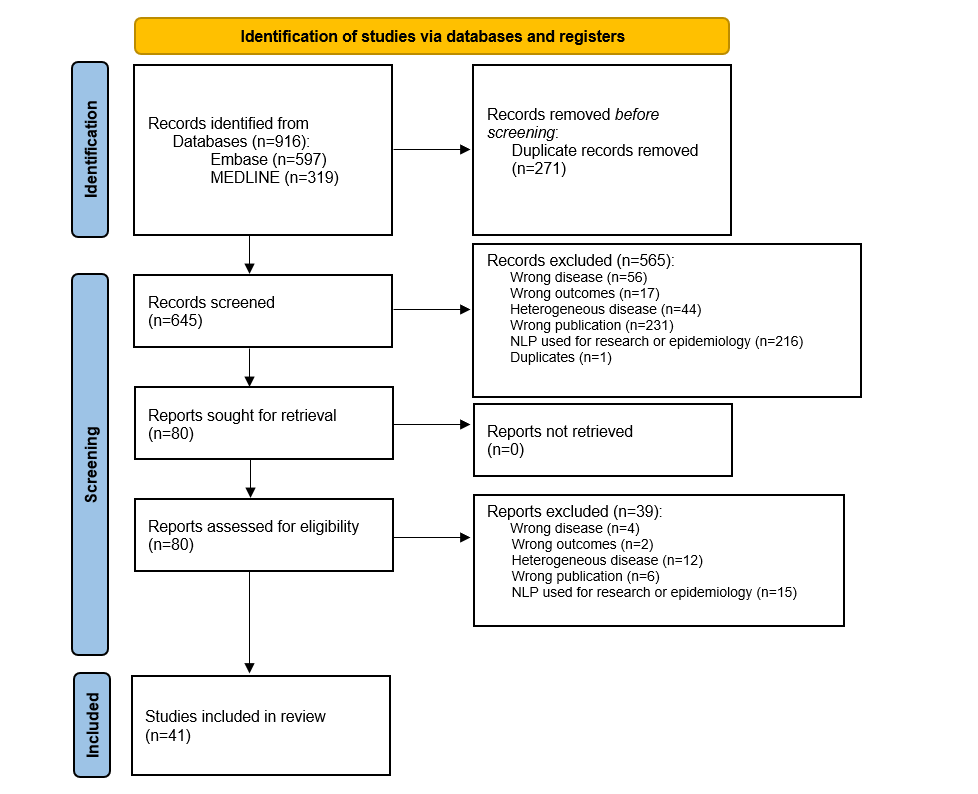
Study PRISMA (Preferred Reporting Items for Systematic Reviews and Meta-Analyses) diagram. NLP: natural language processing.

In total, 916 studies were identified from our search strategy, of which 271 were duplicates and were excluded. We then screened the resulting 645 abstracts, of which 565 were excluded due to not meeting initial eligibility criteria. Of the remaining 80 studies, 39 (49%) were excluded. The 2 most common reasons for exclusion were the use of NLP for nonclinical applications (n=15, 38%) and heterogeneous clinical populations (n=12, 31%). In total, 41 (4.5%) of the 916 studies from the original search results were ultimately included for review (Figure 1 and Table 1).

Of the 41 included studies, NLP was applied to stroke or transient ischemic attack in 20 (49%) studies, epilepsy in 10 (24%) studies, Alzheimer dementia in 6 (15%) studies, and MS in 5 (12%) studies. We found no studies applying NLP to Parkinson disease or migraine that met our eligibility criteria. Across all neurological conditions, NLP was most commonly applied for the purposes of detection or diagnosis (n=20, 49%), followed by clinical disease phenotyping or subtyping (n=17, 41%), prognostication or risk stratification (n=9, 22%), and management or therapy (n=4, 10%; Table 2).

**Table 1:** Included studies.

Paper authors	Publication date	Country	Source text	Journal field	External model validation	Condition being studied	Purpose of NLP^a^	NLP method	Deep learning	Algorithms used	Study outcomes
Miller et al [19]	May 9, 2022	United States	Radiology reports	Clinical neurology	Yes	Stroke	Detection or diagnosis	Rule-based, ML^b^	Yes	Random forest, linear regression, KNN^c^, lasso regression, MLP^d^, transformer	Radiographic complications of ischemic stroke (eg, hemorrhagic transformation)
Lay et al [25]	October 23, 2020	Australia	Clinical notes	Clinical neurology	No	Epilepsy	Detection or diagnosis	ML	No	Latent Dirichlet allocation	Identifying themes in medical records in patients with PNES^e^, congruency of themes
Mayampurath et al [26]	June 24, 2021	United States	Clinical notes	Clinical neurology	No	Stroke	Detection or diagnosis, clinical disease phenotyping or severity	ML	No	SVM^f^, logistic regression	Acute stroke diagnosis, stroke severity and subtypes
Li et al [16]	March 1, 2021	United States	Radiology reports	Neuroradiology	Yes	Stroke	Detection or diagnosis	Rule-based, ML	No	Random forest	Acute or subacute ischemic stroke cases before and during COVID-19
Lineback et al [27]	July 13, 2021	United States	Clinical notes	Clinical neurology	No	Stroke	Prognosis or risk stratification	ML	No	SVM, naïve Bayes, random forest, logistic regression, shallow neural network, lasso regression, ensemble, boosting	30-day stroke readmission, 30-day all-cause readmission
Liu et al [28]	April 13, 2022	China	Speech	Public health	No	Alzheimer disease	Detection or diagnosis	ML	Yes	SVM, random forest, logistic regression, boosting, CNN^g^, transformer	Detection of Alzheimer disease from speech
Mahajan and Baths [29]	February 5, 2021	India	Speech	Nonclinical neuroscience	No	Alzheimer disease	Detection or diagnosis	ML	Yes	CNN, RNN^h^ (LSTM^i^)	Detection of Alzheimer disease from speech
Bacchi et al [30]	February 20, 2022	Australia	Clinical notes	Clinical medicine	No	Stroke	Clinical disease phenotyping or severity	Rule-based, ML	No	Random forest, decision tree, logistic regression, neural network with an unspecified number of layers	Extraction of stroke key performance indicators
Hamid et al [31]	October 14, 2013	United States	Clinical notes	Clinical neurology	No	Epilepsy	Detection or diagnosis	Rule-based, ML	No	Naïve Bayes	Identification of patients with PNES
Yu et al [13]	September 16, 2020	Canada	Radiology reports	Medical informatics	No	Stroke	Detection or diagnosis, clinical disease phenotyping or severity	Rule-based	No	N/A^j^	Identification of the presence and location of vascular occlusions and other stroke-related attributes
Bacchi et al [32]	January 17, 2019	Australia	Clinical notes and radiology reports	Clinical neurology	No	Stroke	Detection or diagnosis	ML	Yes	Random forest, decision tree, CNN, RNN (LSTM)	Determining the cause of TIA^k^-like presentations (cerebrovascular vs noncerebrovascular)
Garg et al [33]	May 15, 2019	United States	Clinical notes and radiology reports	Clinical neurology	No	Stroke	Clinical disease phenotyping or severity	Rule-based, ML	No	SVM, random forest, logistic regression, KNN, boosting, ensemble (stacking logistic regression, extra trees classifier)	Ischemic stroke subtypes
Zhao et al [21]	March 8, 2021	United States	Clinical notes	Medical informatics	Yes	Stroke	Detection or diagnosis, clinical disease phenotyping or severity	Rule-based, ML	No	Random forest, logistic regression	Incidence of stroke, stroke subtypes
Pevy et al [34]	October 1, 2021	United Kingdom	Speech	Clinical neurology	No	Epilepsy	Detection or diagnosis	ML	No	Random forest	Distinguishing between PNES and epilepsy, hesitations and repetitions in descriptions of epileptic seizures versus PNES
Guan et al [35]	December 10, 2020	United States	Echocardiographic reports	Clinical neurology	No	Stroke	Clinical disease phenotyping or severity	Rule-based, ML	No	SVM, random forest, decision tree, logistic regression, KNN	Subtyping and phenotyping cardioembolic stroke
Cui et al [36]	June 26, 2014	United States	Clinical notes	Medical informatics	No	Epilepsy	Clinical disease phenotyping or severity	Rule-based	No	N/A	Epilepsy phenotype extraction with correlated anatomic location
Heo et al [37]	December 16, 2020	South Korea	Radiology reports	Clinical medicine	No	Stroke	Prognosis or risk stratification	ML	Yes	SVM, random forest, decision tree, shallow neural network, lasso regression, CNN, RNN (LSTM), MLP	Prediction of poor stroke outcome
Zanotto et al [38]	November 1, 2021	Brazil	Clinical notes	Medical informatics	No	Stroke	Prognosis or risk stratification, clinical disease phenotyping or severity	Rule-based, ML	Yes	SVM, naïve Bayes, random forest, KNN, CNN, transformer	Prediction of stroke outcome measurements and extraction of patient characteristics
Barbour et al [17]	May 21, 2019	United States	Clinical notes	Clinical neurology	Yes	Epilepsy	Prognosis or risk stratification	Rule-based	No	N/A	Risk factors for SUDEP^l^
Kim et al [39]	February 28, 2019	United States	Radiology reports	Nonclinical neuroscience	No	Stroke	Detection or diagnosis	ML	No	SVM, naïve Bayes, decision tree, logistic regression	Identification of acute ischemic stroke, features of acute ischemic stroke reports versus nonischemic stroke reports
Davis et al [40]	October 22, 2013	United States	Clinical notes, letters, and problem lists	Medical informatics	No	MS^m^	Clinical disease phenotyping or severity	Rule-based	No	N/A	Extraction of clinical traits of patients with MS
Glauser et al [41]	January 22, 2020	United States	Speech	Clinical neurology	No	Epilepsy	Detection or diagnosis	Rule-based, ML	No	SVM	Epilepsy psychiatric comorbidities
Cohen et al [42]	May 22, 2016	United States	Clinical notes	Medical informatics	No	Epilepsy	Prognosis or risk stratification, management or therapy	ML	No	SVM, naïve Bayes	Identification of potential candidates for surgical intervention for pediatric drug–resistant epilepsy, performance of classification algorithm over time
Alim-Marvasti et al [43]	February 10, 2021	United Kingdom	Clinical notes and radiology reports	Medical informatics	No	Epilepsy	Clinical disease phenotyping or severity, prognosis or risk stratification	Rule-based, ML	No	SVM, naïve Bayes, random forest, logistic regression, boosting	Localizing the epileptogenic zone (temporal vs extra-temporal), postsurgical prognosis and outcome
Balagopalan et al [44]	April 27, 2021	Canada	Speech	Nonclinical neuroscience	No	Alzheimer disease	Detection or diagnosis	ML	Yes	SVM, naïve Bayes, random forest, linear regression, shallow neural network, ridge regression, transformer	Detection of Alzheimer disease from speech, prediction of MMSE^n^
Martinc et al [45]	June 14, 2021	Slovenia	Speech	Nonclinical neuroscience	No	Alzheimer disease	Detection or diagnosis	ML	Yes	SVM, random forest, logistic regression, boosting, transformer	Detection of Alzheimer disease from speech
Liu et al [46]	April 5, 2022	United States	Speech	Clinical neurology	No	Alzheimer disease	Detection or diagnosis	ML	Yes	Shallow neural network, transformer	Detection of Alzheimer disease from speech
Nelson et al [47]	December 22, 2016	United States	Clinical notes	Pharmacy	No	MS	Clinical disease phenotyping or severity	Rule-based	No	N/A	Identification of MS phenotype, percentages of each phenotype
Deng et al [18]	April 8, 2022	China	Clinical notes and radiology reports	Nonclinical neuroscience	Yes	Stroke	Management or therapy	Rule-based, ML	Yes	Transformer	Performance of system to generate ICH^o^ treatment plan
Chase et al [48]	February 28, 2017	United States	Clinical notes	Medical informatics	No	MS	Detection or diagnosis	Rule-based, ML	No	Naïve Bayes	Early detection of MS
Wissel et al [49]	November 29, 2019	United States	Clinical notes	Clinical neurology	No	Epilepsy	Prognosis or risk stratification, management or therapy	ML	No	SVM	Epilepsy surgery candidacy score
Sung et al [50]	February 28, 2020	Taiwan	Clinical notes	Medical informatics	No	Stroke	Clinical disease phenotyping or severity	Rule-based, ML	No	SVM, random forest, decision tree, logistic regression, KNN, ensemble	Classification of ischemic stroke subtypes
Sung et al [20]	November 19, 2021	Taiwan	Clinical notes and radiology reports	Clinical neurology	Yes	Stroke	Prognosis or risk stratification	ML	Yes	Random forest, logistic regression, transformer	Prediction of poor functional outcome after acute ischemic stroke
Yang et al [51]	October 20, 2020	Canada	Clinical notes	Medical informatics	No	MS	Clinical disease phenotyping or severity	Rule-based ML	Yes	Shallow neural network, CNN, RNN	Expanded disability status scale score, expanded disability status scale subscore
Xie et al [52]	February 22, 2022	United States	Clinical notes	Medical informatics	No	Epilepsy	Clinical disease phenotyping or severity	ML	Yes	Transformer	Seizure freedom, seizure frequency, date of last seizure
Sung et al [53]	February 8, 2018	Taiwan	Clinical notes	Medical informatics	No	Stroke	Management or therapy	Rule-based	No	N/A	Performance of EMR^p^ interface that determines eligibility for intravenous thrombolytic therapy
Sung et al [54]	February 17, 2022	Taiwan	Clinical notes and radiology reports	Medical informatics	No	Stroke	Prognosis or risk stratification	Rule-based, ML	No	Logistic regression, boosting, unspecified penalized logistic regression method, ensemble (extra trees classifier)	Prediction of poor functional outcome after acute ischemic stroke
Xia et al [55]	November 11, 2013	United States	Clinical notes and radiology reports	Nonclinical neuroscience	No	MS	Detection or diagnosis, clinical disease phenotyping or severity	Rule-based, ML	No	Lasso regression, stepwise regression	Identification of patients with MS, severity of MS
Ong et al [22]	June 19, 2020	United States	Radiology reports	Nonclinical neuroscience	Yes	Stroke	Detection or diagnosis, clinical disease phenotyping or severity	ML	Yes	Random forest, decision tree, logistic regression, KNN, RNN (LSTM)	Ischemic stroke presence, location, and acuity
Roshanzamir et al [56]	March 9, 2021	Iran	Speech	Medical informatics	No	Alzheimer disease	Detection or diagnosis	ML	Yes	Logistic regression, shallow neural network, CNN, RNN (LSTM) transformer	Detection of Alzheimer disease from speech
Rannikmäe et al [57]	June 15, 2021	United Kingdom	Radiology reports	Medical informatics	No	Stroke	Clinical disease phenotyping or severity	Rule-based, ML	Yes	RNN	Stroke subtypes

^a^NLP: natural language processing.

^b^ML: machine learning.

^c^KNN: k-nearest neighbor.

^d^MLP: multilayer perceptron.

^e^PNES: psychogenic nonepileptic seizures.

^f^SVM: support vector machine.

^g^CNN: convolutional neural network.

^h^RNN: recurrent neural network.

^i^LSTM: long- and short-term memory network.

^j^N/A: Not applicable.

^k^TIA: transient ischemic attack.

^l^SUDEP: sudden unexpected death in epilepsy.

^m^MS: multiple sclerosis.

^n^MMSE: Mini-Mental Status Examination.

^o^ICH: intracerebral hemorrhage.

^p^EMR: electronic medical record.

**Table 2:** Overall study characteristics: journal field, target of NLP^a^, and neurological condition.

Study characteristics		Studies (n=41), n (%)
**Condition**		
	Stroke	20 (49)
	Epilepsy	10 (24)
	Alzheimer disease	6 (15)
	Multiple sclerosis	5 (12)
**Target of NLP**		
	Diagnosis	20 (49)
	Phenotyping	17 (42)
	Prognosis	9 (22)
	Therapy	4 (10)
**Journal field**		
	Medical informatics	15 (37)
	Clinical neurology	14 (34)
	Nonclinical neuroscience	7 (17)
	Clinical medicine	2 (5)
	Other^b^	3 (7)

^a^NLP: natural language processing.

^b^Other includes studies published in pharmacy, public health, and neuroradiology journals.

Of the 41 studies, the language sources for NLP comprised clinical notes (n=25, 61%); radiology reports (n=14, 34%); speech (n=8, 20%); and other sources (n=2, 5%) that included echocardiography reports, letters to referring providers, and problem lists (Table 3). Of studies with speech as the language source, half (4/8, 50%) analyzed transcripts only, whereas half additionally incorporated acoustic features from the audio files themselves. These transcripts and audio files were largely from research datasets (eg, ADReSS and Pitt corpus). Two studies analyzed transcripts from interviews with patients. In the study including problem lists, it is unknown who reported the problems.

**Table 3:** Overall study characteristics: NLP^a^ methods and language sources.

Study characteristics		Studies (n=41), n (%)
**NLP method**		
	Rule-based	23 (56)
	Machine learning	35 (85)
**Type of** **machine learning**		
	Conventional machine learning	31 (76)
	Deep learning	16 (39)
**Source text**		
	Clinical notes	25 (61)
	Radiology reports	14 (34)
	Speech	8 (20)
	Other^b^	2 (5)

^a^NLP: natural language processing.

^b^Other includes echocardiography reports, problem lists, and letters to referring providers.

Of the 41 studies, the most common source language for NLP was English (n=39, 95%), Portuguese in 1 (2%) study, and unspecified in the remaining 1 study (which was of Chinese nationality, not multicentric). When patient population size was recorded, the median was 1091 (IQR 188-4211). In studies that did not specify a population size (n=4, 10%), the median number of clinical or radiographic notes was 2172 (IQR 1155.5-22,018.0).

**Figure 2:**
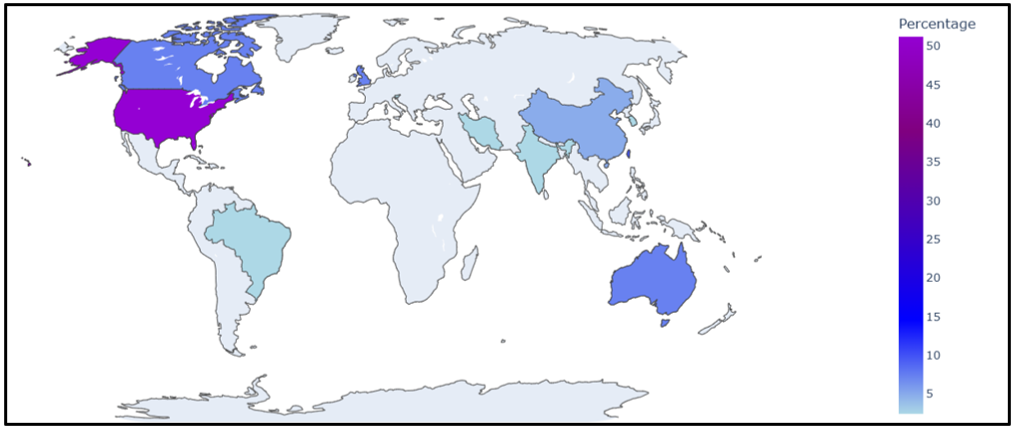
Proportion of included studies (n=41), organized according to country of origin: the United States (n=21, 51%); Taiwan (n=4, 10%); the United Kingdom, Canada, and Australia (n=3, 7% each); China (n=2, 5%); and South Korea, Brazil, Iran, India, and Slovenia (n=1, 2% each).

Papers were most commonly published in medical informatics (n=15, 37%) journals, followed closely by clinical neurology (n=14, 34%) journals. Seven (17%) studies were published in nonclinical neuroscience journals; 2 (5%) in clinical medicine journals; and 1 (2%) each in neuroradiology, public health, and pharmacy journals. Studies were mostly conducted in the United States (n=21, 51%), followed by Taiwan (n=4, 10%) and the United Kingdom, Canada, and Australia (n=3, 7% each). Two (5%) studies were conducted in China, and 1 (2%) study was conducted in each of South Korea, Brazil, Iran, India, and Slovenia (Figure 2).

Only 6 (15%) studies used strictly rule-based methods. The majority of studies incorporated ML (n=35, 85%), either exclusively (n=18, 44%) or in combination with rule-based methods (n=17, 41%). Of the studies that used ML, most (n=31, 89%) used conventional ML methods, whereas 16 (46%) used DL approaches (Table 3), and 12 (34%) used a combination of both conventional ML and DL approaches.

**Figure 3:**
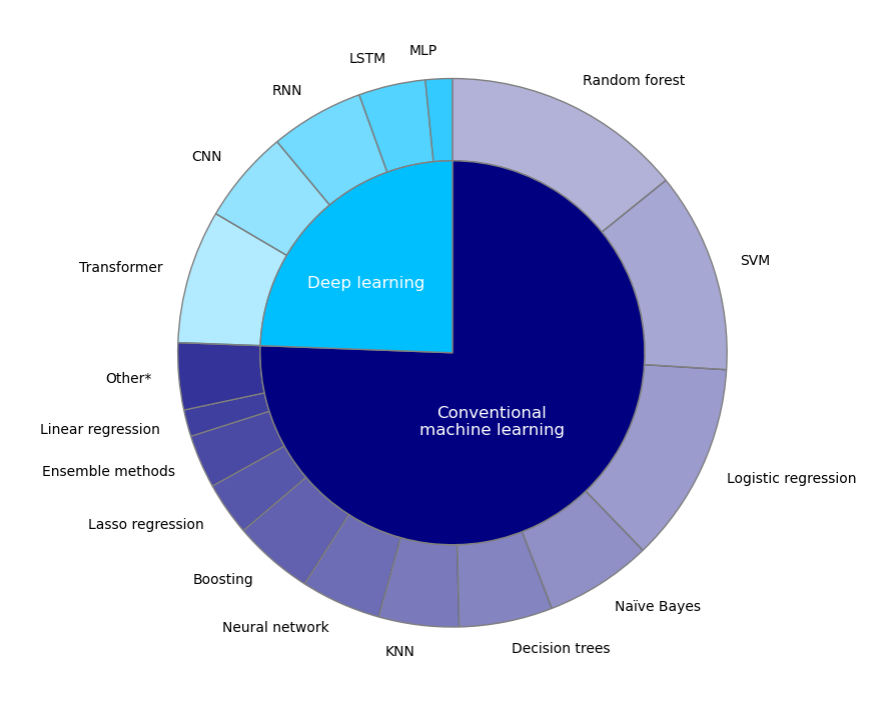
Relative proportions of machine learning algorithms used by the included NLP models. CNN: convolutional neural network; KNN: k-nearest neighbor; LSTM: long- and short-term memory networks; MLP: multilayer perceptron; RNN: recurrent neural network; SVM: support vector machine. *Other includes stepwise regression, ridge regression, an unspecified penalized regression method, latent Dirichlet allocation, and an unspecified neural network with an unspecified number of layers.

**Figure 4:**
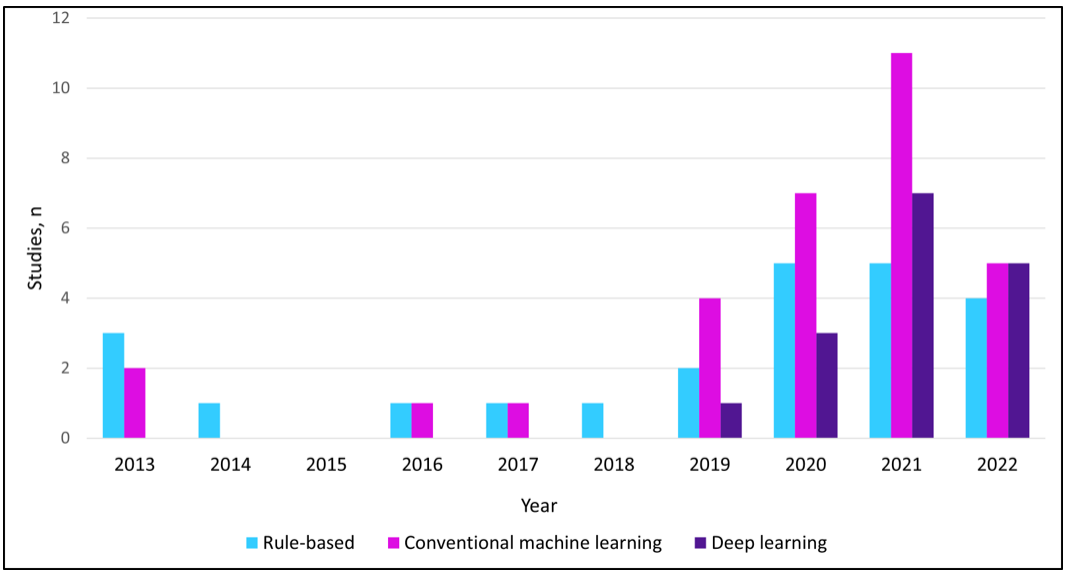
Number of studies applying natural language processing (NLP) to neurological conditions, stratified by NLP methodology and publication year.

As shown in Figure 3, the most frequently used conventional ML algorithms were random forest (n=18, 58%), SVM (n=15, 48%), and logistic regression (n=15, 48%) models. Among studies using DL approaches, transformers (n=10, 63%) were the most commonly used algorithm, followed by convolutional neural networks and RNNs (each n=7, 44%). The co-occurrence of random forest and transformer algorithms was a prevalent trend in research combining traditional ML with DL methodologies (n=6, 15%). Studies that used DL only began to appear in 2019 and later (Figure 4). The most often reported performance metrics for ML models were precision or recall (n=31, 76%), accuracy (n=22, 54%), area under the receiver operating curve (n=20, 49%), and *F*_1_-score (n=19, 46%).

All 41 studies were model derivation studies, with only 7 (17%) studies conducting additional external validation (Multimedia Appendix 2). Furthermore, nearly all the study models were developed retrospectively and were not applied in practice or deployed in real-world environments, except for 3 studies. A study by Li et al [16] developed a model for stroke detection from imaging reports and then applied it to quantify the change in stroke cases before and during the COVID-19 pandemic. A second by Sung et al [53], also in the stroke category, evaluated the deployment of a user-interface system to determine intravenous thrombolysis eligibility built on the NLP model devised. A third study by Wissel et al [49] created a model to identify surgical resection candidates in adult patients with epilepsy. The model was retrained prospectively to incorporate new information.

### Study Characteristics, Stratified by Condition

In studies focused on Alzheimer dementia, diagnosis and detection was the only target of NLP (6/6, 100%). Disease phenotyping and subtyping was the most common purpose of NLP in stroke (10/20, 50%) and MS (4/5, 80%), whereas prognostication was seen as often as diagnosis in epilepsy studies (4/10, 40%; Figure S9 in Multimedia Appendix 2). Studies that applied NLP for the purpose of disease treatment or management were limited to stroke and epilepsy (Figure S9 in Multimedia Appendix 2).

Rule-based methods were used across all studies, except for Alzheimer dementia, in which only ML approaches were used (Figure S10 in Multimedia Appendix 2). Conventional ML methods were used most often by Alzheimer dementia studies (5/6, 83%), followed by stroke (16/20, 80%). Similarly, DL methods were used predominantly by Alzheimer dementia (6/6, 100%) and stroke (8/20, 40%) studies (Figure S10 in Multimedia Appendix 2). The transformer was the DL method used most frequently in Alzheimer disease-related studies (5/6, 83%).

## Discussion

### Principal Findings

In this scoping review, 41 studies [13,16-22,25-57] that investigated direct clinical applications of NLP to common neurological disorders were identified. We found that the majority of these studies focused on detection and diagnosis and applied NLP to stroke, whereas we found no studies of NLP that met our eligibility criteria in the clinical areas of migraine or Parkinson disease. Methodologically, ML techniques were used more often than rule-based methods, but a considerable number of studies still relied on rule-based approaches in combination with ML. While we observed that DL began to emerge as a methodology for NLP in 2019, we found that the transformer was the most commonly used DL algorithm overall.

At the time of writing, we believe our scoping review to be the first to examine direct clinical NLP applications in common neurological conditions. One prior review [58] investigated NLP applications across the combined clinical specialties of neurosurgery, spine surgery, and neurology, whereas another evaluated the use of NLP in both psychiatry and clinical neuroscience [59]. However, neither reviews analyzed studies and NLP applications according to neurological condition. More importantly, these reviews included many studies where NLP was not applied for direct clinical use, instead aiming to perform tasks such as characterizing patient cohorts [58], analyzing information extraction, or determining causal inference between concepts [59]. In contrast to this prior work, our review focused on direct clinical applications of NLP.

Of note, we found no studies applying NLP to migraine or Parkinson disease that met our eligibility criteria, thereby highlighting a potential gap in NLP research focusing on these disorders. This is perhaps unexpected, as the combined prevalence of migraine and Parkinson disease in the United States exceeds that of both stroke and MS [12]. Two explanations may account for this finding. One is that migraine and Parkinson disease may rely less on radiographic imaging studies and their reports to establish a diagnosis than stroke, Alzheimer dementia, or MS. Given that many ML applications in stroke have focused on neuroimaging [60], it is plausible that stroke imaging reports could represent an important source of data for NLP analyses. Indeed, the results of our review demonstrate that stroke-related NLP studies made use of radiographic reports as often as clinical notes for source text, which could have resulted in a relatively higher number of NLP studies within stroke than in other neurological conditions.

A second explanation may be that Alzheimer disease is a more common cause of dementia worldwide than dementing syndromes associated with Parkinson disease [61] and has in turn garnered a larger proportion of research funding. National Institutes of Health [62] research funding for Alzheimer dementia was approximately US $3 billion in 2022, as compared to US $259 million for Parkinson disease.

Our finding that NLP was most frequently applied to diagnostic problems is expected, given that clinical decision support is a common focus of artificial intelligence in medicine [63]. Historically, clinical decision support has also played an important role in medical informatics by constituting the main focus of archetypal systems such as MYCIN, INTERNIST-1, and DXplain, which were first developed in the 1970s and 1980s [64]. An alternative explanation is that the shortage of neurologists that already exists worldwide [65] may have potentially created a more urgent need for detection-oriented NLP applications rather than NLP applications targeting therapeutic management or prognostication.

Though diagnosis was the most common target of NLP overall, we found that epilepsy-related studies focused as much on prognostication as they did on diagnostic tasks. Given that roughly one-third of all patients with epilepsy are drug resistant [66], determining good surgical resection candidates as well as predicting surgical outcomes are important objectives that have been the focus of considerable research [67]. Consistent with this, the epilepsy-related studies in the prognostication category were directed toward identifying adult [49] and pediatric [42] surgical candidates, predicting postsurgical outcomes [43], and detecting risk factors for sudden unexpected death in epilepsy [17].

With respect to the types of ML models we found in our review, the relatively high proportion of conventional ML-based studies using random forest and SVM (18/31, 58% and 15/31, 48%, respectively) may have been related to the fact that SVM together with random forest models generally represented the dominant ML techniques prior to the advent of neural networks [68] in diagnostic and clinical decision support applications [63,69,70]. Despite its position as a potentially more basic classification method than either SVM or random forest, logistic regression was used as commonly as SVM in our analysis.

Furthermore, while we found that SVM and random forest models were common in ML-based NLP approaches, the optimal problems these models address are fundamentally different. SVM generally works best as a binary classifier, whereas random forest models are best used for classification tasks involving multiple categories [71]. We found that the most frequently used ML algorithms in stroke-related NLP studies were random forest models. This matches the most frequent target of NLP in stroke-related studies, which was disease subtyping (a multiple classification problem).

Among DL algorithms, which are becoming increasingly widespread in NLP [72], the transformer was the most commonly used technique we identified. Unlike other word embedding methods, a transformer processes a whole sequence of text while preserving the context and meaning of words [59,73]. Another significant advantage of transformers is that they can use transfer learning, which first trains a model on a learning task and then applies the model to a separate but closely related task [58,74]. A prevalent example of transfer learning in our results is Bidirectional Encoder Representations From Transformers (BERT), a transformer model that was originally trained using publicly available text from Wikipedia and BookCorpus, a collection of free, unpublished novels consisting of over 50 million sentences [75,76]. BERT can then be further refined on a target training task and dataset before being passed to a separate classification algorithm [28]. This is helpful in situations where the target training set is small [28]. The high frequency of Alzheimer disease–related NLP studies we found using BERT is expected within this context, as these studies often used the ADReSS speech dataset that consists of only 78 healthy controls and 78 patients with Alzheimer disease [28,45].

A particularly important finding of our review is that although many of the NLP studies leveraged powerful and sophisticated computational tools, most studies constitute research work rather than reports of operationalization or evaluation in practical settings. This is consistent with the current state of clinical NLP outside of neurology, wherein real-world deployment of NLP models continues to be limited [7,77,78].

One major obstacle to the implementation of NLP in clinical practice is model generalizability [7]. Published NLP models are usually internally validated rather than externally validated [7,17], limiting the understanding of model accuracy beyond the model’s original training environment [60]. We found this to be true for the majority of studies identified in our review. The lack of EMR standardization, including note formatting [17,78], documentation styles, and radiographic report structures across different medical institutions [7] and between clinicians, may partly account for our observations. Furthermore, the preponderance of English language as source text in NLP [79], as demonstrated by the single study in our review using non-English (Portuguese) text for analysis, suggests that the generalizability of NLP within neurology is most likely limited outside the English language.

Another major obstacle impeding the adoption of NLP tools is the inherent lack of transparency of ML-based algorithms [60], particularly artificial neural networks and other forms of DL approaches [80]. These approaches have low transparency because the computational methods they use to characterize relationships between inputs and outputs are not readily intelligible to humans [7,78,80] acting as a black box that could undermine clinicians’ trust in their performance.

The lack of well-defined regulatory guidelines and standards overseeing the artificial intelligence space [81] has furthered this mistrust. Compromise of personal health data, algorithmic bias, and the question of how to attribute culpability when diagnostic errors arise [82,83] are all ethical concerns that may serve to explain the relative paucity of studies across all neurological conditions that externally validated DL models.

Finally, the lack of portability of NLP applications into external EMRs is another factor that has restricted the development of NLP models to the research arena. External software modules containing ML and DL models are challenging to integrate into EMRs [1,84], as most implementations require a high level of computing infrastructure and technical expertise that many hospital information technology systems and personnel may lack [84]. Recent work suggests few EMR-integrated aggregative tools exist to display NLP findings to clinicians in a digestible format [85]. To address these barriers, some authors have advocated for collaborations between NLP researchers and EMR companies [77].

### Limitations and Future Work

Our scoping review has several limitations. First, we note that the target of NLP was categorized according to author experience and interpretation of the literature, which may have underreported the application of the published NLP algorithms. Second, due to the variable performance metrics and outcomes across studies, we did not aggregate measurements of performance in our review, and we therefore could not reliably provide summary performance metrics for NLP models within individual diseases, applications, or outcomes. Future work should focus on individual outcomes within a clinical disorder for a more exact appraisal of NLP model performance than this review.

Third, this review only included studies based on common neurological disorders, direct clinical applications of NLP, and homogeneous clinical populations, which limited the number of studies we identified. It is therefore important to note that this review cannot be used to make definitive conclusions on the state of NLP research across all neurological disorders. Future efforts can be directed at characterizing the use of NLP across less common neurological disorders as well as in heterogeneous or ambiguously defined clinical populations. As NLP technologies continue to advance, it will also be critically important to evaluate studies that use newer transformers, such as GPT3, which have better performance than BERT models [59].

### Conclusions

The abundance of unstructured text data in modern-day EMRs as well as the emphasis in neurology on narrative history and physical examination and heavy reliance on ancillary information such as radiographic reports and speech, all create an optimal use case for applying NLP for the diagnosis, management, or prognostication of neurological disorders. To our knowledge, this is the first attempt to systematically characterize research efforts to investigate direct NLP applications to common neurological conditions. Our review reveals gaps in neurological NLP research, showing a relative deficiency of NLP studies in subspecialties outside of stroke or epilepsy, and underlines the need to actualize NLP models outside of the research phase. Moreover, the current emphasis of NLP on diagnostic tasks suggests that NLP may be particularly useful in settings that lack access to neurological expertise.

## Appendix

Multimedia Appendix 1 PRISMA-ScR (Preferred Reporting Items for Systematic Reviews and Meta-Analyses Extension for Scoping Reviews): checklist and explanation.

Multimedia Appendix 2 Search strategy and additional data.

## Abbreviations

BERT: Bidirectional Encoder Representations From Transformers
DL: deep learning
EMR: electronic medical record
ML: machine learning
MS: multiple sclerosis
NLP: natural language processing
PRISMA-ScR: Preferred Reporting Items for Systematic Reviews and Meta-Analyses Extension for Scoping Reviews
PROSPERO: Prospective Register of Systematic Reviews
REDCap: Research Electronic Data Capture
RNN: recurrent neural network
SVM: support vector machine

## References

[1] Pivovarov R, Elhadad N (2015). Automated methods for the summarization of electronic health records. J Am Med Inform Assoc.

[2] Locke S, Bashall A, Al-Adely S, Moore J, Wilson A, Kitchen GB (2021). Natural language processing in medicine: a review. Trends Anaesth Crit Care.

[3] Kimia AA, Savova G, Landschaft A, Harper MB (2015). An introduction to natural language processing: how you can get more from those electronic notes you are generating. Pediatr Emerg Care.

[4] Iroju OG, Olaleke JO (2015). A systematic review of natural language processing in healthcare. Int J Inf Technol Comput Sci.

[5] Wang J, Deng H, Liu B, Hu A, Liang J, Fan L, Zheng X, Wang T, Lei J (2020). Systematic evaluation of research progress on natural language processing in medicine over the past 20 years: bibliometric study on PubMed. J Med Internet Res.

[6] Gunter D, Puac-Polanco P, Miguel O, Thornhill RE, Yu AYX, Liu ZA, Mamdani M, Pou-Prom C, Aviv RI (2022). Rule-based natural language processing for automation of stroke data extraction: a validation study. Neuroradiology.

[7] Pons E, Braun LMM, Hunink MGM, Kors JA (2016). Natural language processing in radiology: a systematic review. Radiology.

[8] Ohno-Machado L, Nadkarni P, Johnson K (2013). Natural language processing: algorithms and tools to extract computable information from EHRs and from the biomedical literature. J Am Med Inform Assoc.

[9] Zhou B, Yang G, Shi Z, Ma S (2024). Natural language processing for smart healthcare. IEEE Rev Biomed Eng.

[10] Le Glaz A, Haralambous Y, Kim-Dufor DH, Lenca P, Billot R, Ryan TC, Marsh J, DeVylder J, Walter M, Berrouiguet S, Lemey C (2021). Machine learning and natural language processing in mental health: systematic review. J Med Internet Res.

[11] Chin JH, Vora N (2014). The global burden of neurologic diseases. Neurology.

[12] Gooch CL, Pracht E, Borenstein AR (2017). The burden of neurological disease in the United States: a summary report and call to action. Ann Neurol.

[13] Yu AYX, Liu ZA, Pou-Prom C, Lopes K, Kapral MK, Aviv RI, Mamdani M (2021). Automating stroke data extraction from free-text radiology reports using natural language processing: instrument validation study. JMIR Med Inform.

[14] Pinter NK, Fritz JV (2020). Neuroimaging for the neurologist: clinical MRI and future trends. Neurol Clin.

[15] de la Fuente Garcia S, Ritchie CW, Luz S (2020). Artificial intelligence, speech, and language processing approaches to monitoring Alzheimer's disease: a systematic review. J Alzheimers Dis.

[16] Li MD, Lang M, Deng F, Chang K, Buch K, Rincon S, Mehan WA, Leslie-Mazwi TM, Kalpathy-Cramer J (2021). Analysis of stroke detection during the COVID-19 pandemic using natural language processing of radiology reports. AJNR Am J Neuroradiol.

[17] Barbour K, Hesdorffer DC, Tian N, Yozawitz EG, McGoldrick PE, Wolf S, McDonough TL, Nelson A, Loddenkemper T, Basma N, Johnson SB, Grinspan ZM (2019). Automated detection of sudden unexpected death in epilepsy risk factors in electronic medical records using natural language processing. Epilepsia.

[18] Deng B, Zhu W, Sun X, Xie Y, Dan W, Zhan Y, Xia Y, Liang X, Li J, Shi Q, Jiang L (2022). Development and validation of an automatic system for intracerebral hemorrhage medical text recognition and treatment plan output. Front Aging Neurosci.

[19] Miller MI, Orfanoudaki A, Cronin M, Saglam H, So Yeon Kim I, Balogun O, Tzalidi M, Vasilopoulos K, Fanaropoulou G, Fanaropoulou NM, Kalin J, Hutch M, Prescott BR, Brush B, Benjamin EJ, Shin M, Mian A, Greer DM, Smirnakis SM, Ong CJ (2022). Natural language processing of radiology reports to detect complications of ischemic stroke. Neurocrit Care.

[20] Sung SF, Chen CH, Pan RC, Hu YH, Jeng JS (2021). Natural language processing enhances prediction of functional outcome after acute ischemic stroke. JAHA.

[21] Zhao Y, Fu S, Bielinski SJ, Decker PA, Chamberlain AM, Roger VL, Liu H, Larson NB (2021). Natural language processing and machine learning for identifying incident stroke from electronic health records: algorithm development and validation. J Med Internet Res.

[22] Ong CJ, Orfanoudaki A, Zhang R, Caprasse FPM, Hutch M, Ma L, Fard D, Balogun O, Miller MI, Minnig M, Saglam H, Prescott B, Greer DM, Smirnakis S, Bertsimas D (2020). Machine learning and natural language processing methods to identify ischemic stroke, acuity and location from radiology reports. PLoS One.

[23] Skansi S (2018). Introduction to Deep Learning: From Logical Calculus to Artificial Intelligence. 1st Edition.

[24] Mikolov T, Chen K, Corrado G, Dean J Efficient estimation of word representations in vector space. ArXiv. Preprint posted online on January 16, 2013.

[25] Lay J, Seneviratne U, Fok A, Roberts H, Phan T (2020). Discovering themes in medical records of patients with psychogenic non-epileptic seizures. BMJ Neurol Open.

[26] Mayampurath A, Parnianpour Z, Richards CT, Meurer WJ, Lee J, Ankenman B, Perry O, Mendelson SJ, Holl JL, Prabhakaran S (2021). Improving prehospital stroke diagnosis using natural language processing of paramedic reports. Stroke.

[27] Lineback CM, Garg R, Oh E, Naidech AM, Holl JL, Prabhakaran S (2021). Prediction of 30-day readmission after stroke using machine learning and natural language processing. Front Neurol.

[28] Liu N, Luo K, Yuan Z, Chen Y (2022). A transfer learning method for detecting Alzheimer's disease based on speech and natural language processing. Front Public Health.

[29] Mahajan P, Baths V (2021). Acoustic and language based deep learning approaches for Alzheimer's Dementia detection from spontaneous speech. Front Aging Neurosci.

[30] Bacchi S, Gluck S, Koblar S, Jannes J, Kleinig T (2022). Automated information extraction from free-text medical documents for stroke key performance indicators: a pilot study. Intern Med J.

[31] Hamid H, Fodeh SJ, Lizama AG, Czlapinski R, Pugh MJ, LaFrance WC, Brandt CA (2013). Validating a natural language processing tool to exclude psychogenic nonepileptic seizures in electronic medical record-based epilepsy research. Epilepsy Behav.

[32] Bacchi S, Oakden-Rayner L, Zerner T, Kleinig T, Patel S, Jannes J (2019). Deep learning natural language processing successfully predicts the cerebrovascular cause of transient ischemic attack-like presentations. Stroke.

[33] Garg R, Oh E, Naidech A, Kording K, Prabhakaran S (2019). Automating ischemic stroke subtype classification using machine learning and natural language processing. J Stroke Cerebrovasc Dis.

[34] Pevy N, Christensen H, Walker T, Reuber M (2021). Feasibility of using an automated analysis of formulation effort in patients' spoken seizure descriptions in the differential diagnosis of epileptic and nonepileptic seizures. Seizure.

[35] Guan W, Ko D, Khurshid S, Trisini Lipsanopoulos AT, Ashburner JM, Harrington LX, Rost NS, Atlas SJ, Singer DE, McManus DD, Anderson CD, Lubitz SA (2021). Automated electronic phenotyping of cardioembolic stroke. Stroke.

[36] Cui L, Sahoo SS, Lhatoo SD, Garg G, Rai P, Bozorgi A, Zhang GQ (2014). Complex epilepsy phenotype extraction from narrative clinical discharge summaries. J Biomed Inform.

[37] Heo TS, Kim YS, Choi JM, Jeong YS, Seo SY, Lee JH, Jeon JP, Kim C (2020). Prediction of stroke outcome using natural language processing-based machine learning of radiology report of brain MRI. J Pers Med.

[38] Zanotto BS, da Silva Etges APB, Dal Bosco A, Cortes EG, Ruschel R, De Souza AC, Andrade CMV, Viegas F, Canuto S, Luiz W, Martins SO, Vieira R, Polanczyk C, Gonçalves MA (2021). Stroke outcome measurements from electronic medical records: cross-sectional study on the effectiveness of neural and nonneural classifiers. JMIR Med Inform.

[39] Kim C, Zhu V, Obeid J, Lenert L (2019). Natural language processing and machine learning algorithm to identify brain MRI reports with acute ischemic stroke. PLoS One.

[40] Davis MF, Sriram S, Bush WS, Denny JC, Haines JL (2013). Automated extraction of clinical traits of multiple sclerosis in electronic medical records. J Am Med Inform Assoc.

[41] Glauser T, Santel D, DelBello M, Faist R, Toon T, Clark P, McCourt R, Wissel B, Pestian J (2020). Identifying epilepsy psychiatric comorbidities with machine learning. Acta Neurol Scand.

[42] Cohen KB, Glass B, Greiner HM, Holland-Bouley K, Standridge S, Arya R, Faist R, Morita D, Mangano F, Connolly B, Glauser T, Pestian J (2016). Methodological issues in predicting pediatric epilepsy surgery candidates through natural language processing and machine learning. Biomed Inform Insights.

[43] Alim-Marvasti A, Pérez-García F, Dahele K, Romagnoli G, Diehl B, Sparks R, Ourselin S, Clarkson MJ, Duncan JS (2021). Machine learning for localizing epileptogenic-zone in the temporal lobe: quantifying the value of multimodal clinical-semiology and imaging concordance. Front Digit Health.

[44] Balagopalan A, Eyre B, Robin J, Rudzicz F, Novikova J (2021). Comparing pre-trained and feature-based models for prediction of Alzheimer's disease based on speech. Front Aging Neurosci.

[45] Martinc M, Haider F, Pollak S, Luz S (2021). Temporal integration of text transcripts and acoustic features for Alzheimer's diagnosis based on spontaneous speech. Front Aging Neurosci.

[46] Liu Z, Paek EJ, Yoon SO, Casenhiser D, Zhou W, Zhao X (2022). Detecting Alzheimer's disease using natural language processing of referential communication task transcripts. J Alzheimers Dis.

[47] Nelson RE, Butler J, LaFleur J, Knippenberg K, Kamauu AWC, DuVall SL (2016). Determining multiple sclerosis phenotype from electronic medical records. J Manag Care Spec Pharm.

[48] Chase HS, Mitrani LR, Lu GG, Fulgieri DJ (2017). Early recognition of multiple sclerosis using natural language processing of the electronic health record. BMC Med Inform Decis Mak.

[49] Wissel BD, Greiner HM, Glauser TA, Holland-Bouley KD, Mangano FT, Santel D, Faist R, Zhang N, Pestian JP, Szczesniak RD, Dexheimer JW (2020). Prospective validation of a machine learning model that uses provider notes to identify candidates for resective epilepsy surgery. Epilepsia.

[50] Sung SF, Lin CY, Hu YH (2020). EMR-based phenotyping of ischemic stroke using supervised machine learning and text mining techniques. IEEE J Biomed Health Inform.

[51] Yang Z, Pou-Prom C, Jones A, Banning M, Dai D, Mamdani M, Oh J, Antoniou T (2022). Assessment of natural language processing methods for ascertaining the expanded disability status scale score from the electronic health records of patients with multiple sclerosis: algorithm development and validation study. JMIR Med Inform.

[52] Xie K, Gallagher RS, Conrad EC, Garrick CO, Baldassano SN, Bernabei JM, Galer PD, Ghosn NJ, Greenblatt AS, Jennings T, Kornspun A, Kulick-Soper CV, Panchal JM, Pattnaik AR, Scheid BH, Wei D, Weitzman M, Muthukrishnan R, Kim J, Litt B, Ellis CA, Roth D (2022). Extracting seizure frequency from epilepsy clinic notes: a machine reading approach to natural language processing. J Am Med Inform Assoc.

[53] Sung SF, Chen K, Wu DP, Hung LC, Su YH, Hu YH (2018). Applying natural language processing techniques to develop a task-specific EMR interface for timely stroke thrombolysis: a feasibility study. Int J Med Inform.

[54] Sung SF, Hsieh CY, Hu YH (2022). Early prediction of functional outcomes after acute ischemic stroke using unstructured clinical text: retrospective cohort study. JMIR Med Inform.

[55] Xia Z, Secor E, Chibnik LB, Bove RM, Cheng S, Chitnis T, Cagan A, Gainer VS, Chen PJ, Liao KP, Shaw SY, Ananthakrishnan AN, Szolovits P, Weiner HL, Karlson EW, Murphy SN, Savova GK, Cai T, Churchill SE, Plenge RM, Kohane IS, De Jager PL (2013). Modeling disease severity in multiple sclerosis using electronic health records. PLoS One.

[56] Roshanzamir A, Aghajan H, Baghshah MS (2021). Transformer-based deep neural network language models for Alzheimer's disease risk assessment from targeted speech. BMC Med Inform Decis Mak.

[57] Rannikmäe K, Wu H, Tominey S, Whiteley W, Allen N, Sudlow C, Biobank Uk (2021). Developing automated methods for disease subtyping in UK Biobank: an exemplar study on stroke. BMC Med Inform Decis Mak.

[58] Buchlak Q, Esmaili N, Bennett C, Farrokhi F (2022). Natural language processing applications in the clinical neurosciences: a machine learning augmented systematic review. Acta Neurochir Suppl.

[59] Crema C, Attardi G, Sartiano D, Redolfi A (2022). Natural language processing in clinical neuroscience and psychiatry: a review. Front Psychiatry.

[60] Wang W, Kiik M, Peek N, Curcin V, Marshall IJ, Rudd AG, Wang Y, Douiri A, Wolfe CD, Bray B (2020). A systematic review of machine learning models for predicting outcomes of stroke with structured data. PLoS One.

[61] Weller J, Budson A (2018). Current understanding of Alzheimer's disease diagnosis and treatment. F1000Res.

[62] Estimates of funding for various Research, Condition, and Disease Categories (RCDC). National Institutes of Health.

[63] Ahsan MM, Luna SA, Siddique Z (2022). Machine-learning-based disease diagnosis: a comprehensive review. Healthcare (Basel).

[64] Kaul V, Enslin S, Gross SA (2020). History of artificial intelligence in medicine. Gastrointest Endosc.

[65] Burton A (2018). How do we fix the shortage of neurologists?. Lancet Neurol.

[66] Löscher W, Potschka H, Sisodiya SM, Vezzani A (2020). Drug resistance in epilepsy: clinical impact, potential mechanisms, and new innovative treatment options. Pharmacol Rev.

[67] Dlugos DJ (2001). The early identification of candidates for epilepsy surgery. Arch Neurol.

[68] Myszczynska MA, Ojamies PN, Lacoste AMB, Neil D, Saffari A, Mead R, Hautbergue GM, Holbrook JD, Ferraiuolo L (2020). Applications of machine learning to diagnosis and treatment of neurodegenerative diseases. Nat Rev Neurol.

[69] Jain V, Chatterjee JM (2020). Machine Learning with Health Care Perspective: Machine Learning and Healthcare. 1st Edition.

[70] Ortiz-Posadas MR (2020). Pattern Recognition Techniques Applied to Biomedical Problems. 1st Edition.

[71] Gray KR, Aljabar P, Heckemann RA, Hammers A, Rueckert D, Alzheimer's Disease Neuroimaging Initiative (2013). Random forest-based similarity measures for multi-modal classification of Alzheimer's disease. Neuroimage.

[72] Sorin V, Barash Y, Konen E, Klang E (2020). Deep learning for natural language processing in radiology—fundamentals and a systematic review. J Am Coll Radiol.

[73] Amrutha K, Prabu P (2022). Effortless and beneficial processing of natural languages using transformers. J Discret Math Sci Cryptogr.

[74] Weiss K, Khoshgoftaar TM, Wang D (2016). A survey of transfer learning. J Big Data.

[75] Devlin J, Chang MW, Lee K, Toutanova K BERT: pre-training of deep bidirectional transformers for language understanding. ArXiv. Preprint posted online on October 11, 2018.

[76] Russell D, Li L, Tian F (2019). Generating text using generative adversarial networks and quick-thought vectors.

[77] Demner-Fushman D, Elhadad N (2016). Aspiring to unintended consequences of natural language processing: a review of recent developments in clinical and consumer-generated text processing. Yearb Med Inform.

[78] Bitterman DS, Miller TA, Mak RH, Savova GK (2021). Clinical natural language processing for radiation oncology: a review and practical primer. Int J Radiat Oncol Biol Phys.

[79] Magueresse A, Carles V, Heetderks E Low-resource languages: a review of past work and future challenges. ArXiv. Preprint posted online on June 12, 2020.

[80] Sajda P (2006). Machine learning for detection and diagnosis of disease. Annu Rev Biomed Eng.

[81] Khan B, Fatima H, Qureshi A, Kumar S, Hanan A, Hussain J, Abdullah S (2023). Drawbacks of artificial intelligence and their potential solutions in the healthcare sector. Biomed Mater Devices.

[82] Habli I, Lawton T, Porter Z (2020). Artificial intelligence in health care: accountability and safety. Bull World Health Organ.

[83] Amann J, Jotterand F, Ienca M (2021). Machine learning (ML) in stroke medicine: opportunities and challenges for risk prediction and prevention. Artificial Intelligence in Brain and Mental Health: Philosophical, Ethical & Policy Issues.

[84] Elbattah M, Arnaud É, Gignon M, Dequen G (2021). The role of text analytics in healthcare: a review of recent developments and applications. https://www.scitepress.org/PublishedPapers/2021/104145/104145.pdf.

[85] Chard K, Russell M, Lussier YA, Mendonça EA, Silverstein JC (2011). A cloud-based approach to medical NLP. AMIA Annu Symp Proc.

