# Supplementary material for: Direct Clinical Applications of Natural Language Processing in Common Neurological Disorders: Scoping Review
Source: JMIR Neurotechnol. 2024 May 22;3:e51822. doi: 10.2196/51822 (PMC12671331; doi:10.2196/51822)
Supplement: Multimedia Appendix 2 [file neuro_v3i1e51822_app2.docx]

Search Criteria

**Ovid EMBASE**

1. exp natural language processing/

2. NLP.ti.

3. natural language process*.tw,kw.

4. 1 or 2 or 3

5. text mining.tw,kw.

6. 4 or 5

7. multiple sclerosis/

8. (multiple* adj3 sclerosis).tw,kw.

9. Alzheimer disease/

10. alzheimer*.tw,kw.

11. Parkinson disease/

12. parkinson*.tw,kw.

13. exp cerebrovascular disease/

14. (cerebrovasc* adj3 (disease* or accident* or disorder*)).tw,kw.

15. ((brain* or cerebr* or transient*) adj4 (isch?emi* or infarct*)).tw,kw.

16. ((brain* or cerebr* or intracerebr* or intracranial* or intraventricular* or subarachnoid* or infarct*) adj4 (h?ematoma* or h?emorrhag*)).tw,kw.

17. stroke*.tw,kw.

18. exp "seizure, epilepsy and convulsion"/

19. (seizure* or epilep* or convulsion* or eclampsia*).tw,kw.

20. (lennox-gastaut or (lennox* adj3 gastaut)).tw,kw.

21. (west* adj3 syndrome*).tw,kw.

22. (infantil* adj3 spasm*).tw,kw.

23. (earl* adj3 myoclon* adj3 encephalopath*).tw,kw.

24. (dravet* or rasmussen*).tw,kw.

25. exp migraine/

26. migrain*.tw,kw.

27. 7 or 8 or 9 or 10 or 11 or 12 or 13 or 14 or 15 or 16 or 17 or 18 or 19 or 20 or 21 or 22 or 23 or 24 or 25 or 26

28. 6 and 27

29. information extraction.tw,kw.

30. medical language processing.tw,kw.

31. 29 or 30

32. 6 or 31

33. 27 and 32

**Ovid MEDLINE**

1. exp natural language processing/

2. NLP.ti.

3. natural language process*.tw,kf.

4. text mining.tw,kw.

5. information extraction.tw,kf.

6. medical language processing.tw,kf.

7. exp Multiple Sclerosis/

8. (multiple* adj3 sclerosis).tw,kw,kf.

9. Alzheimer Disease/

10. alzheimer*.tw,kf.

11. exp Parkinsonian Disorders/

12. parkinson*.tw,kf.

13. exp Cerebrovascular Disorders/

14. (cerebrovasc* adj3 (disease* or accident* or disorder*)).tw,kw,kf.

15. ((brain* or cerebr* or transient*) adj4 (isch?emi* or infarct*)).tw,kw,kf.

16. ((brain* or cerebr* or intracerebr* or intracranial* or intraventricular* or subarachnoid* or infarct*) adj4 (h?ematoma* or h?emorrhag*)).tw,kw,kf.

17. stroke*.tw,kf.

18. exp Seizures/ or exp Epilepsy/ or Eclampsia/

19. (seizure* or epilep* or convulsion* or eclampsia*).tw,kf.

20. (lennox-gastaut or (lennox* adj3 gastaut)).tw,kf.

21. (west* adj3 syndrome*).tw,kw,kf.

22. (infantil* adj3 spasm*).tw,kw,kf.

23. (earl* adj3 myoclon* adj3 encephalopath*).tw,kw,kf.

24. (dravet* or rasmussen*).tw,kf.

25. exp Migraine Disorders/

26. migrain*.tw,kf.

27. 7 or 8 or 9 or 10 or 11 or 12 or 13 or 14 or 15 or 16 or 17 or 18 or 19 or 20 or 21 or 22 or 23 or 24 or 25 or 26

28. 1 or 2 or 3 or 4 or 5 or 6

29. 27 and 28

Figure S1. Stroke Study Characteristics: Journal Field and Target of NLP


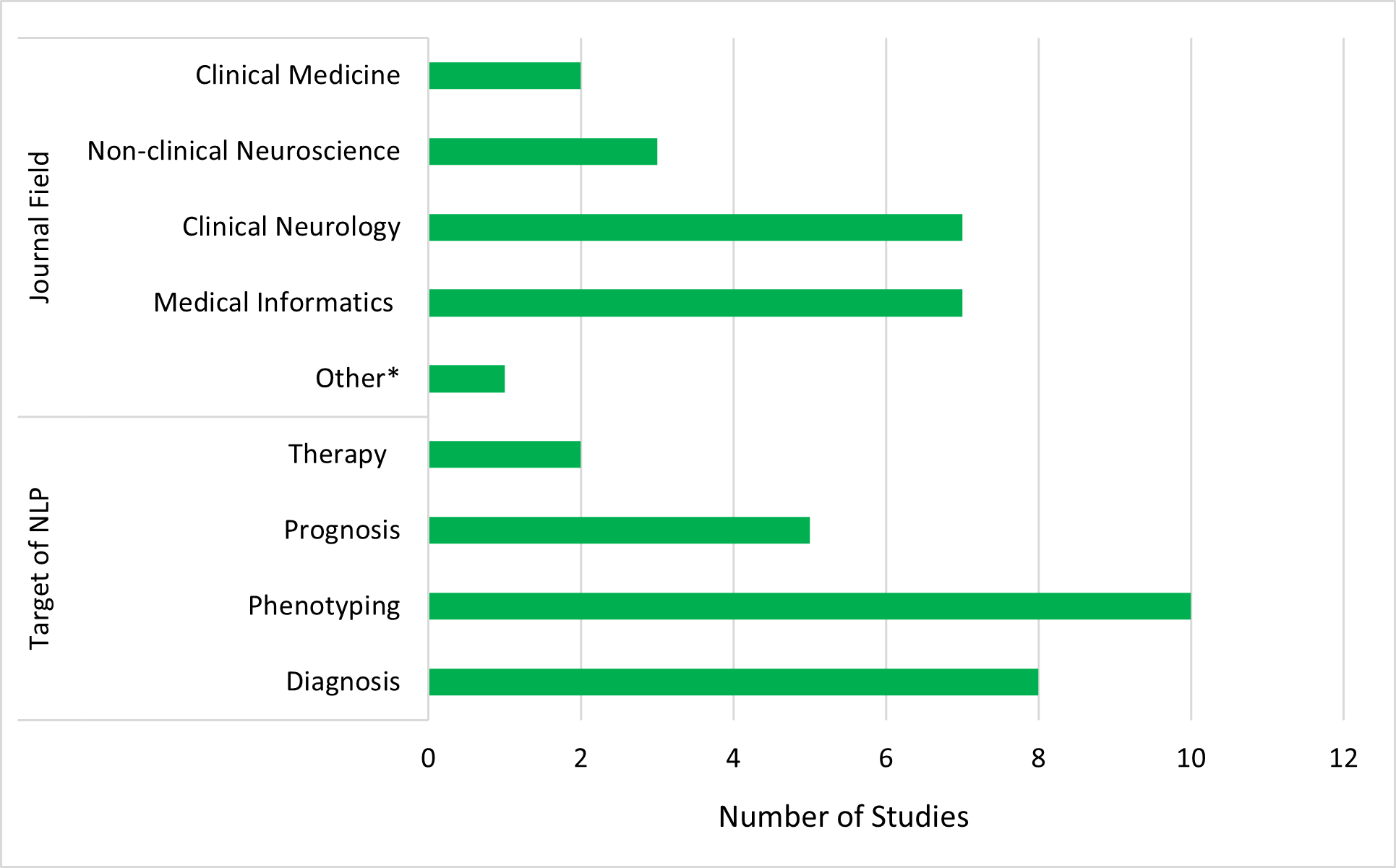


* Other is a neuroradiology journal.

Figure S2. Stroke Study Characteristics: NLP Methods and Language Sources


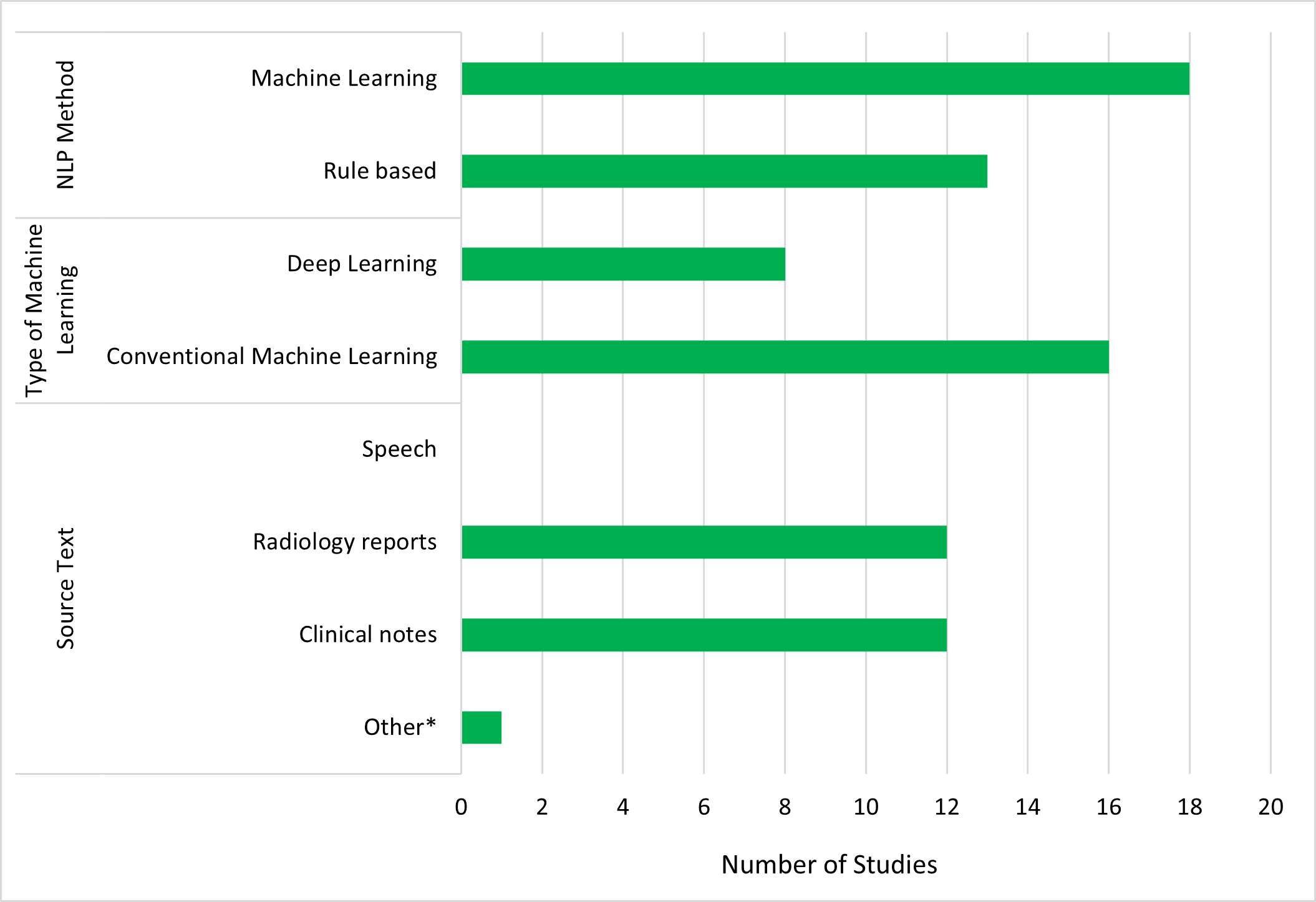


*Other includes echocardiography reports.

Figure S3. Epilepsy Study Characteristics: Journal Field and Target of NLP


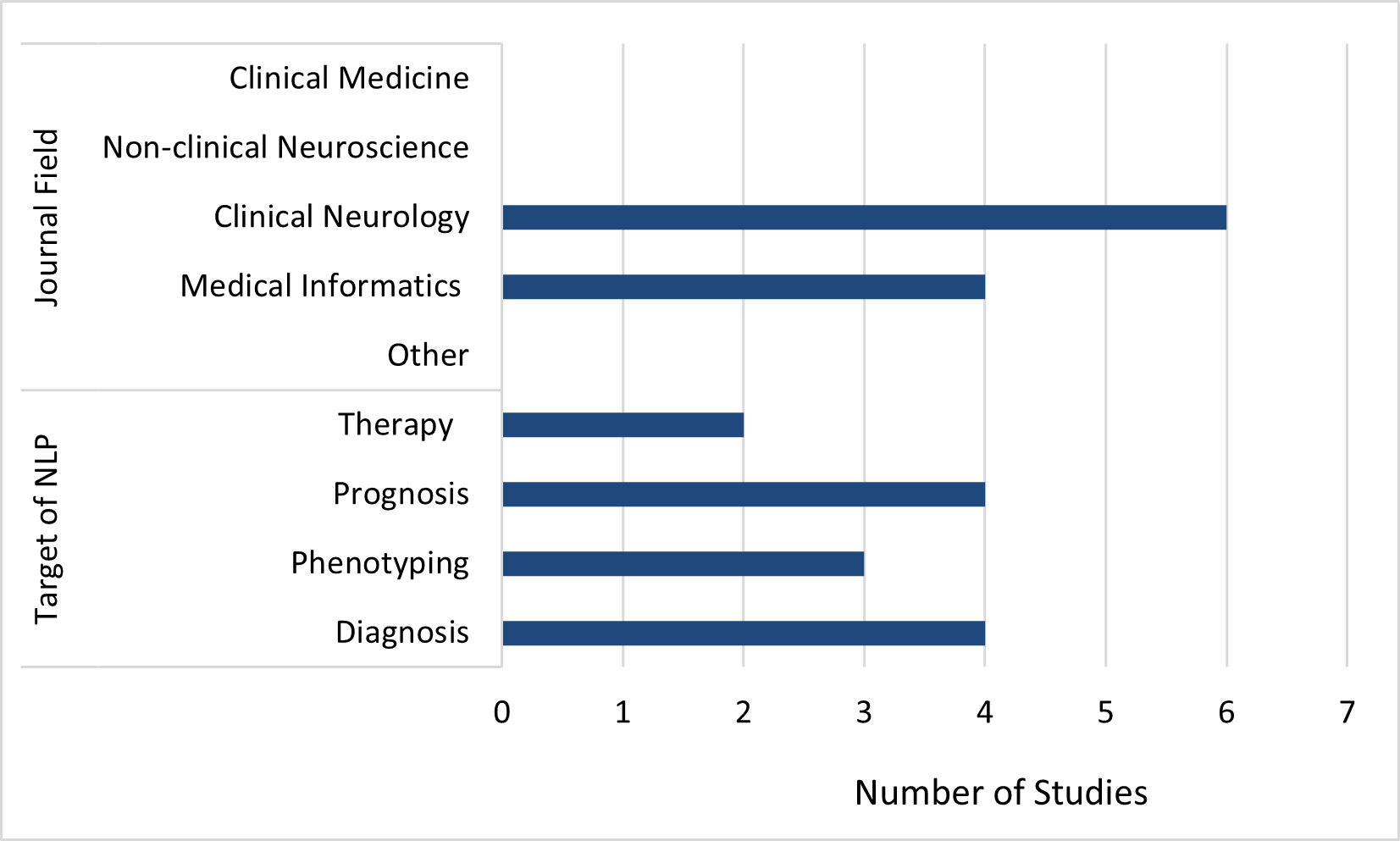


Figure S4. Epilepsy Study Characteristics: NLP Methods and Language Sources


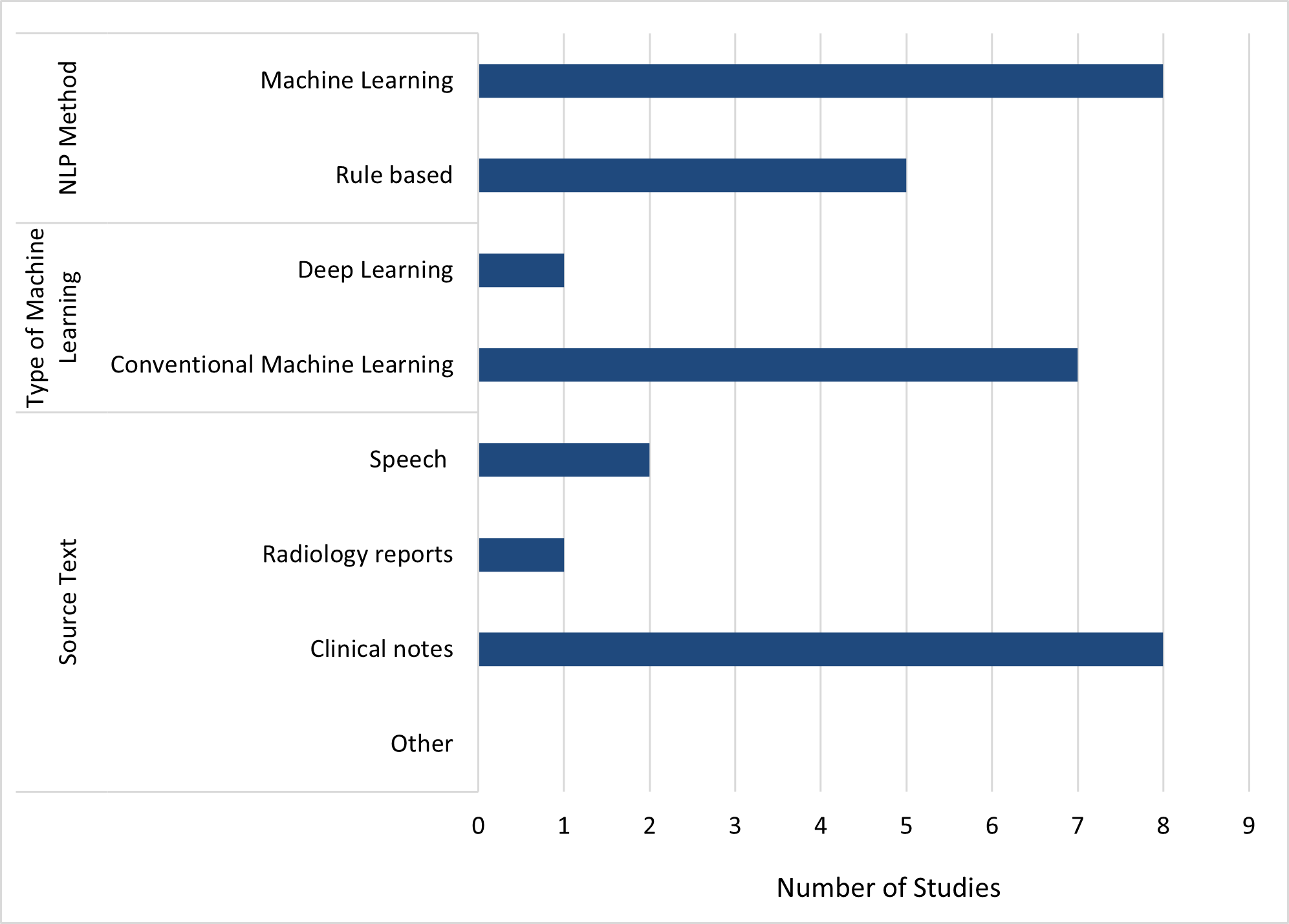


Figure S5. Multiple Sclerosis Study Characteristics: Journal Field and Target of NLP


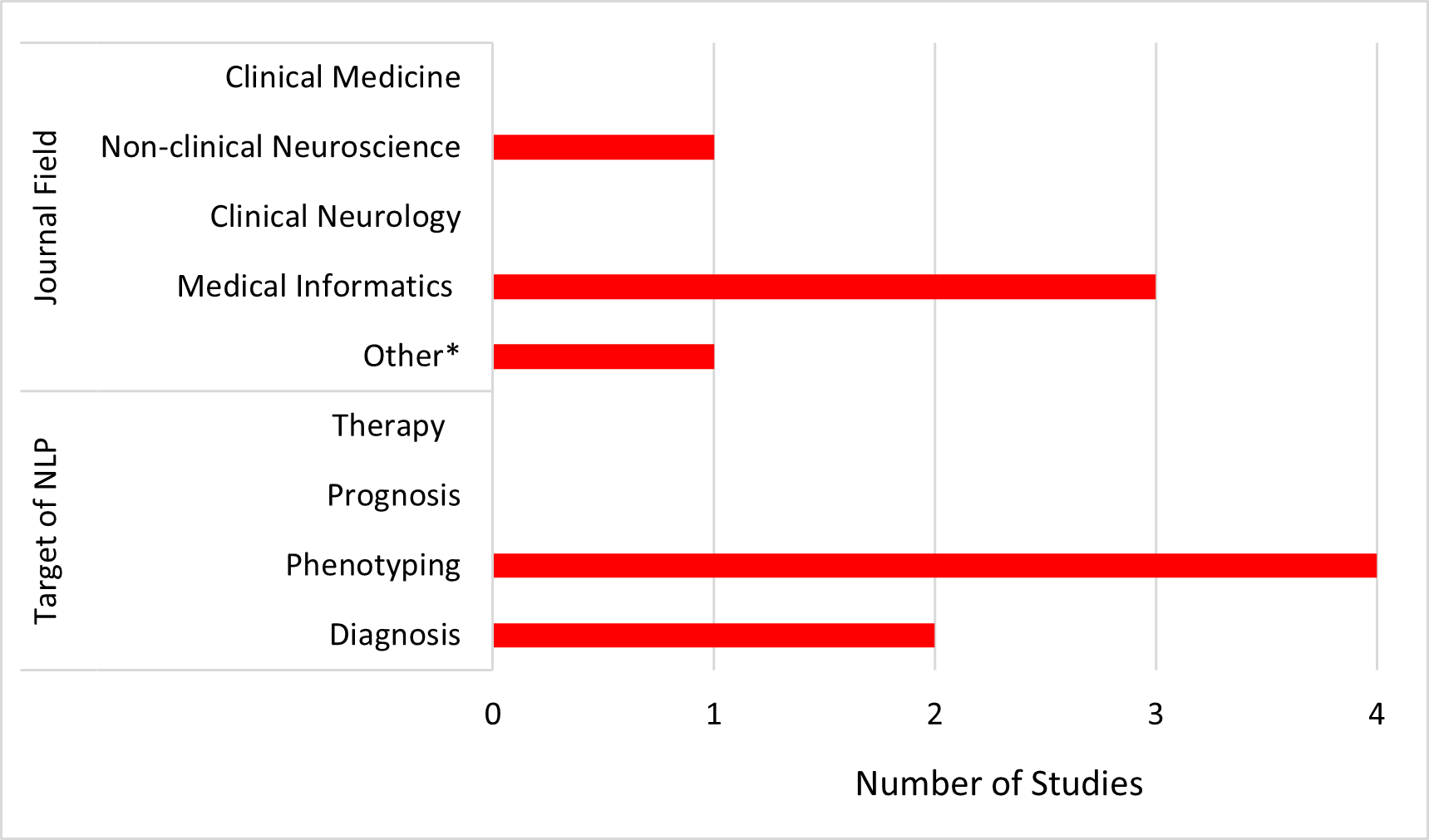


*Other includes a pharmacy journal.

Figure S6. Multiple Sclerosis Study Characteristics: NLP Methods and Language Sources


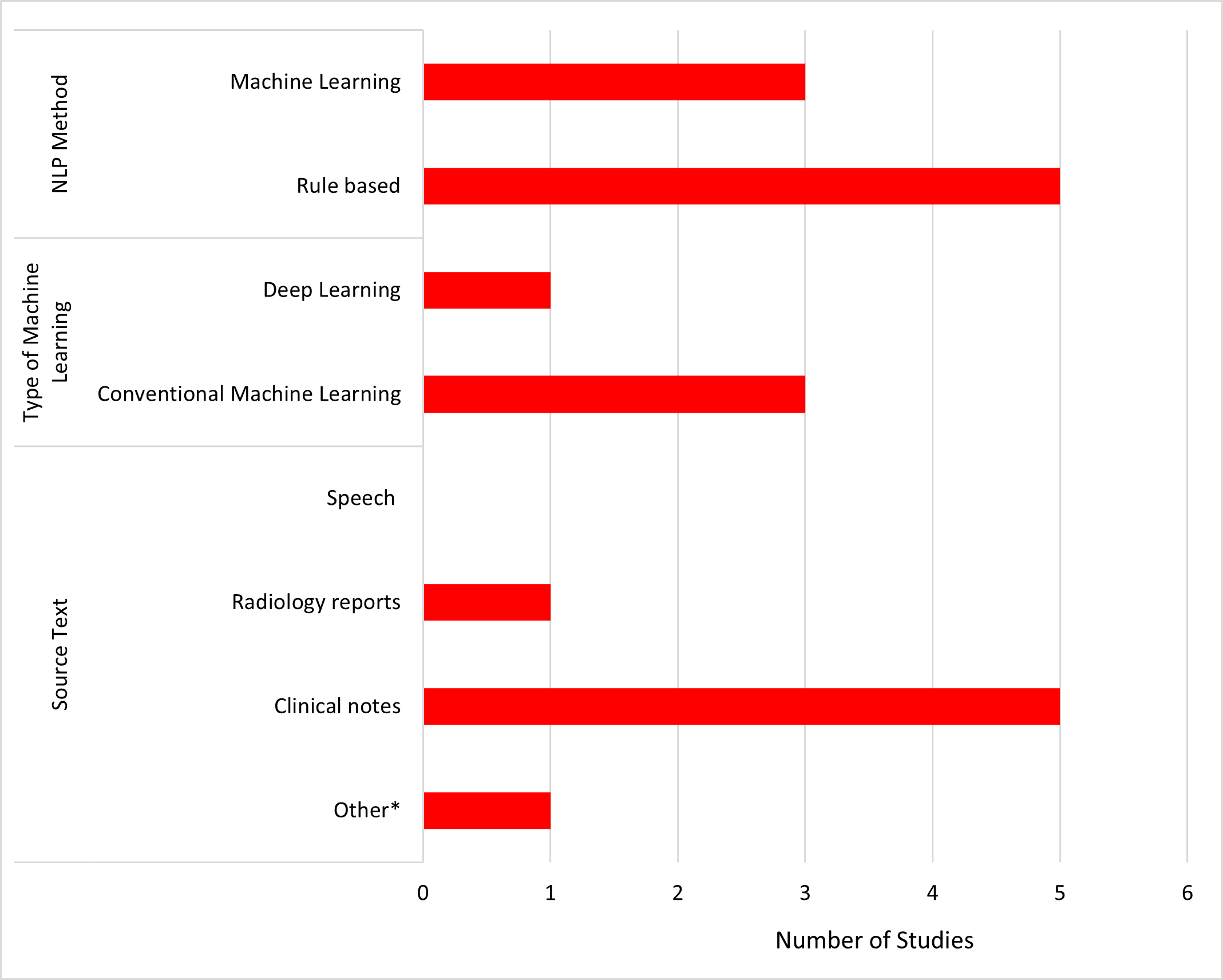


*Other includes letters from referring physicians and problem lists.

Figure S7. Alzheimer Disease Study Characteristics: Journal Field and Target of NLP


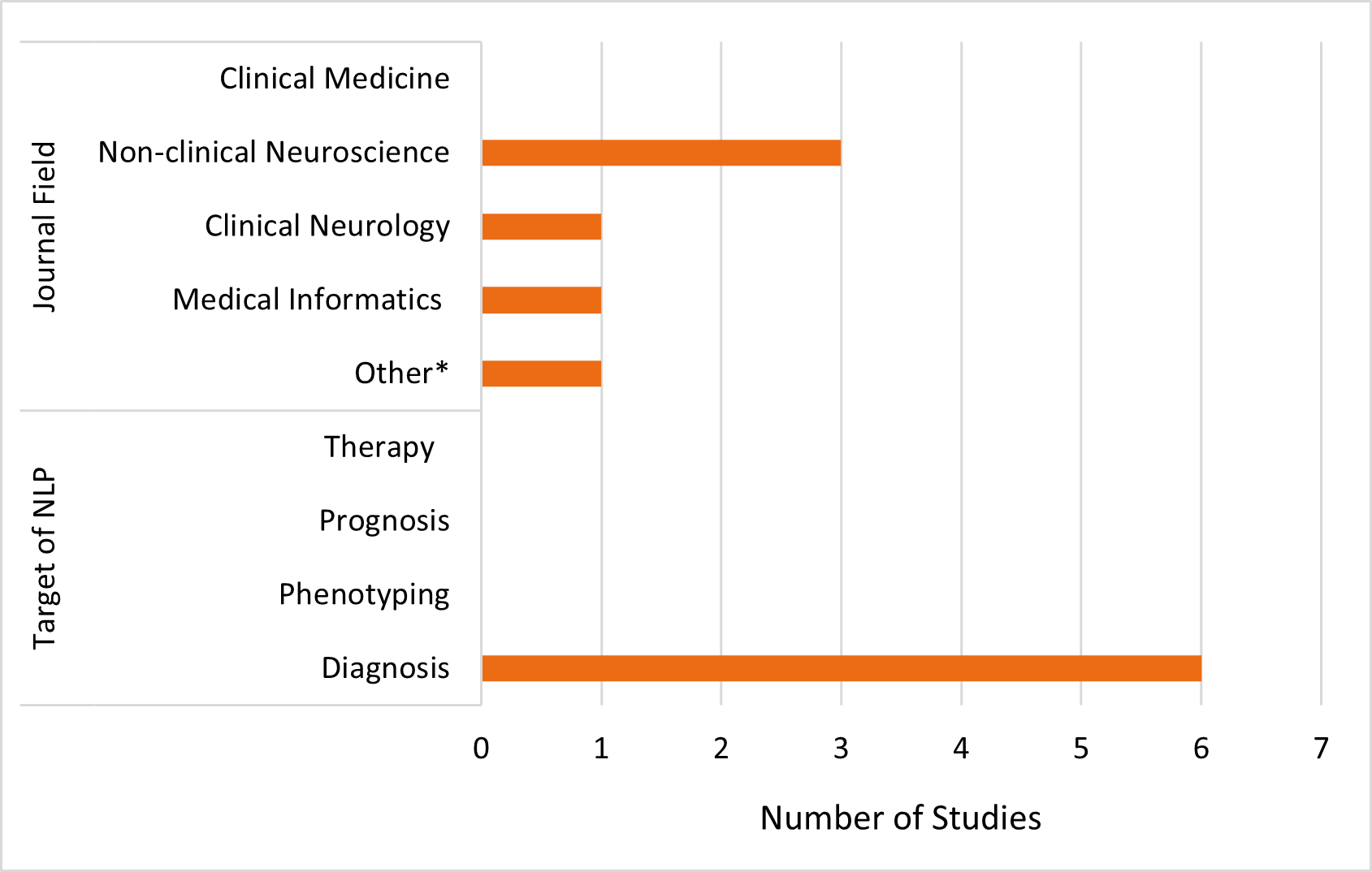


*Other contains one public health journal.

Figure S8. Alzheimer Disease Study Characteristics: NLP Methods and Language Sources


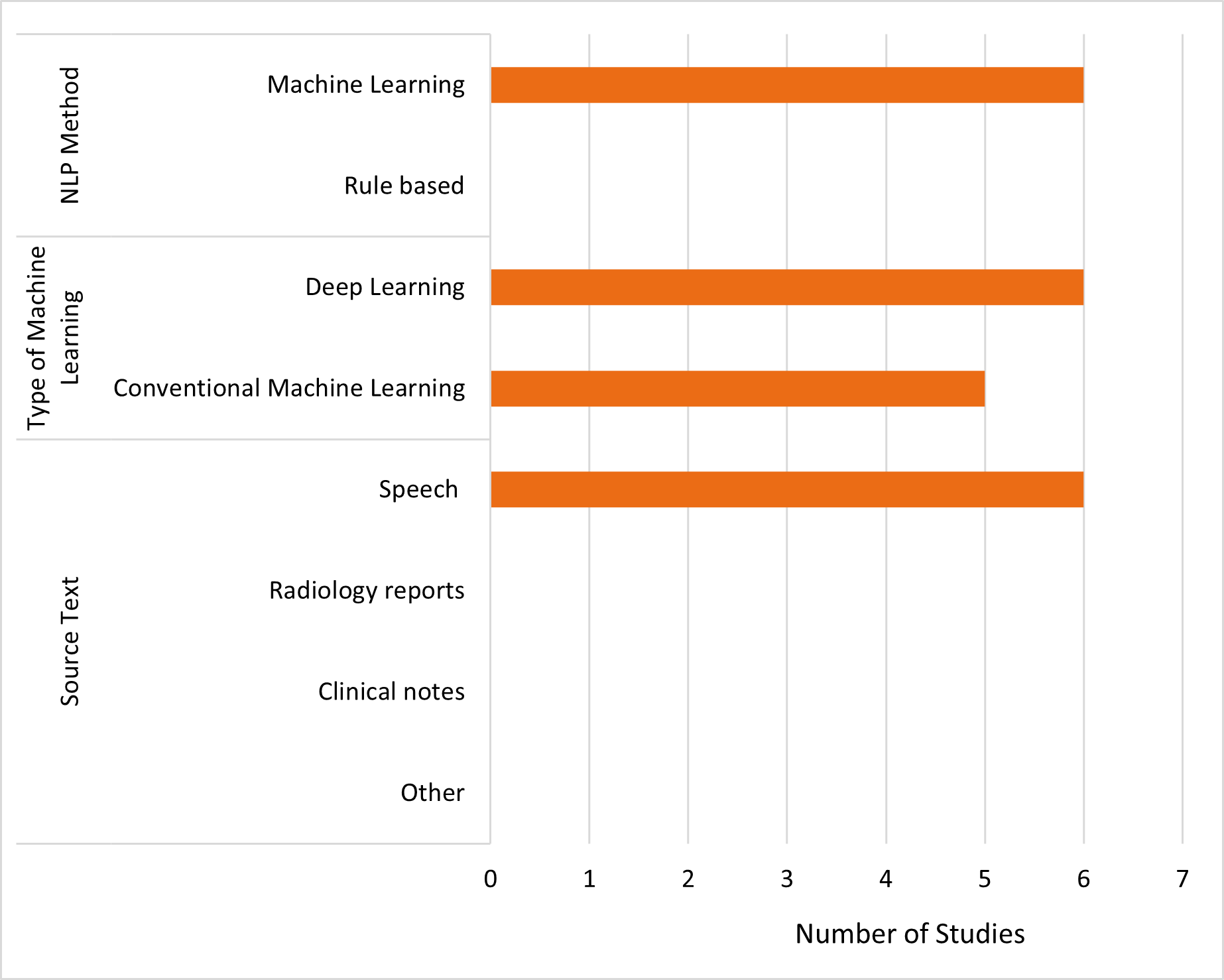


Figure S9. Target of NLP in Studies Represented in the Review, Stratified by Neurological Condition


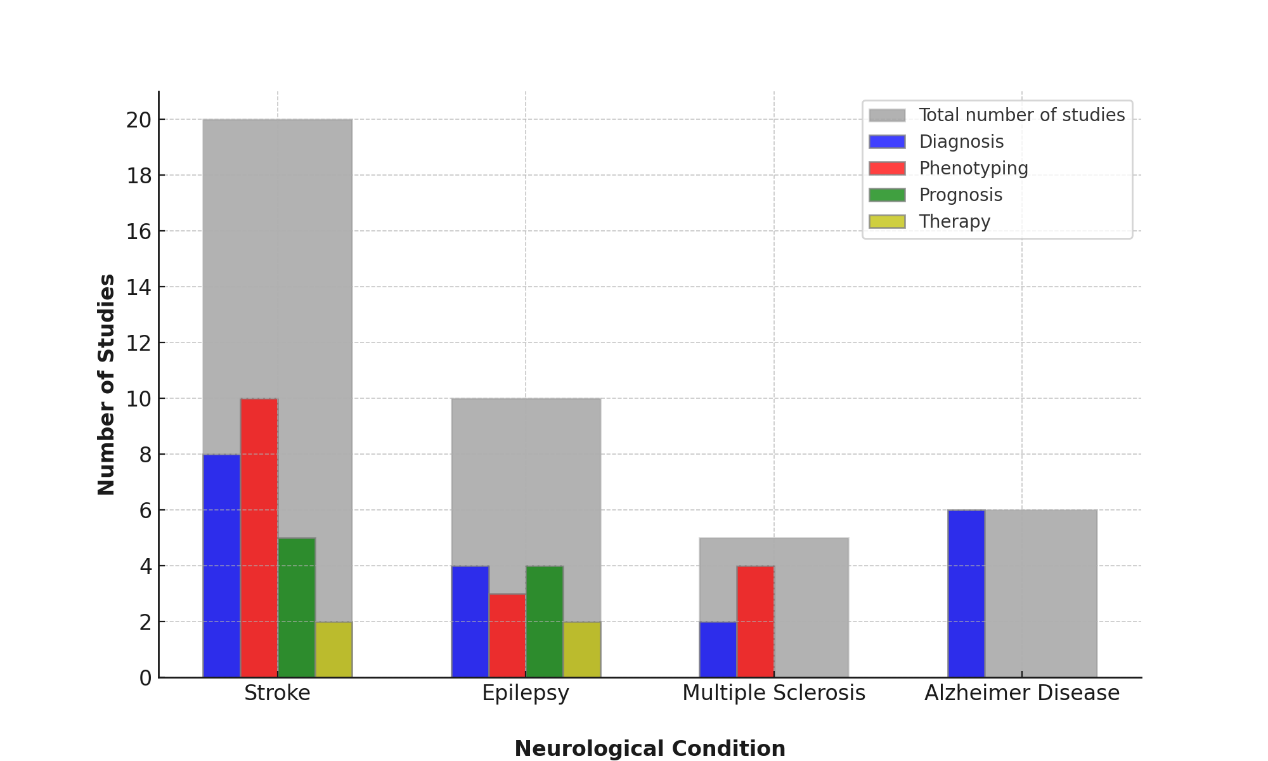


Total number of studies is the N number of studies extracted for the condition.

Figure S10. NLP Methods in Studies Represented in the Review, Stratified by Neurological Condition


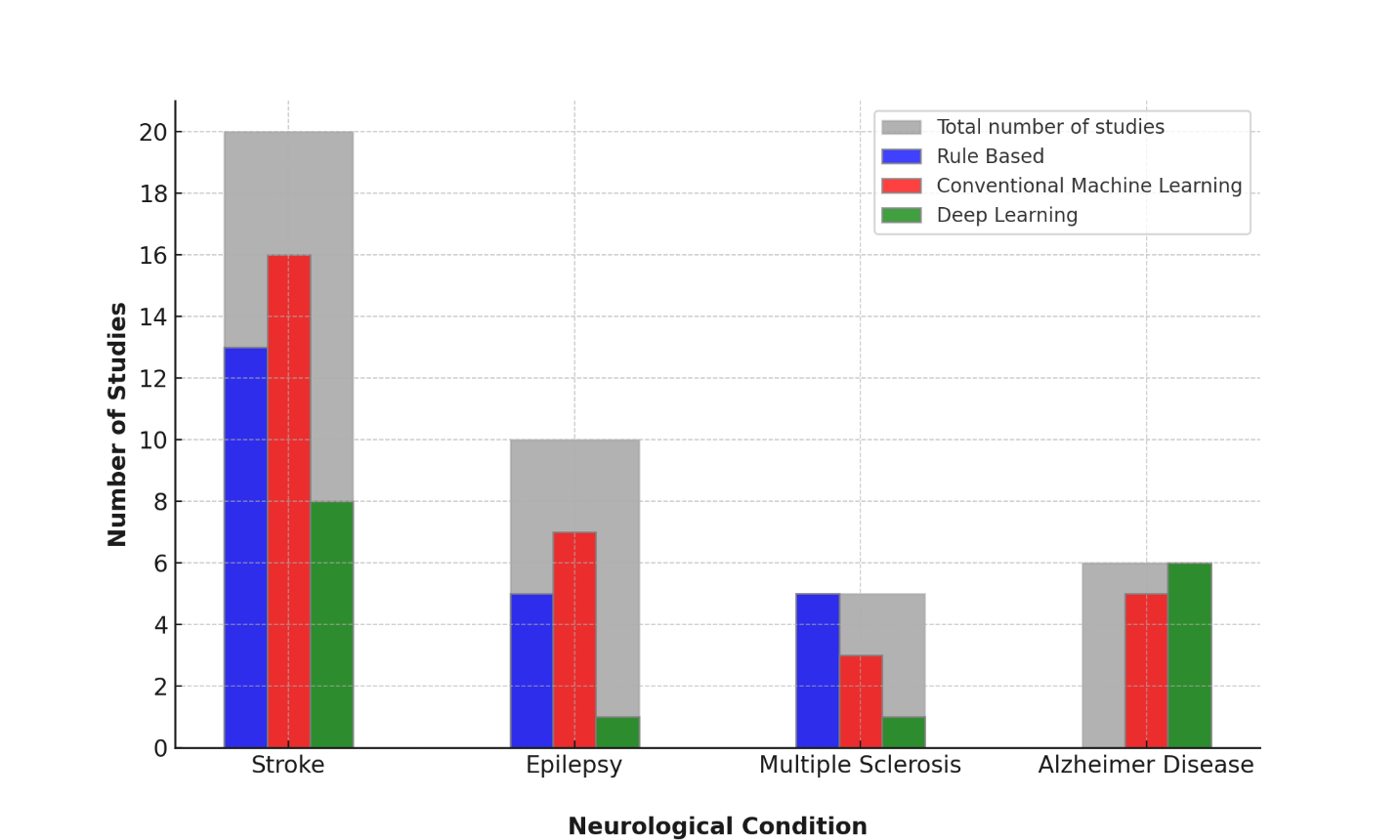


Total number of studies is the N number of studies extracted for the condition.

Figure S11. Relative Proportions of Machine Learning NLP Algorithms Used for Detection/Diagnosis


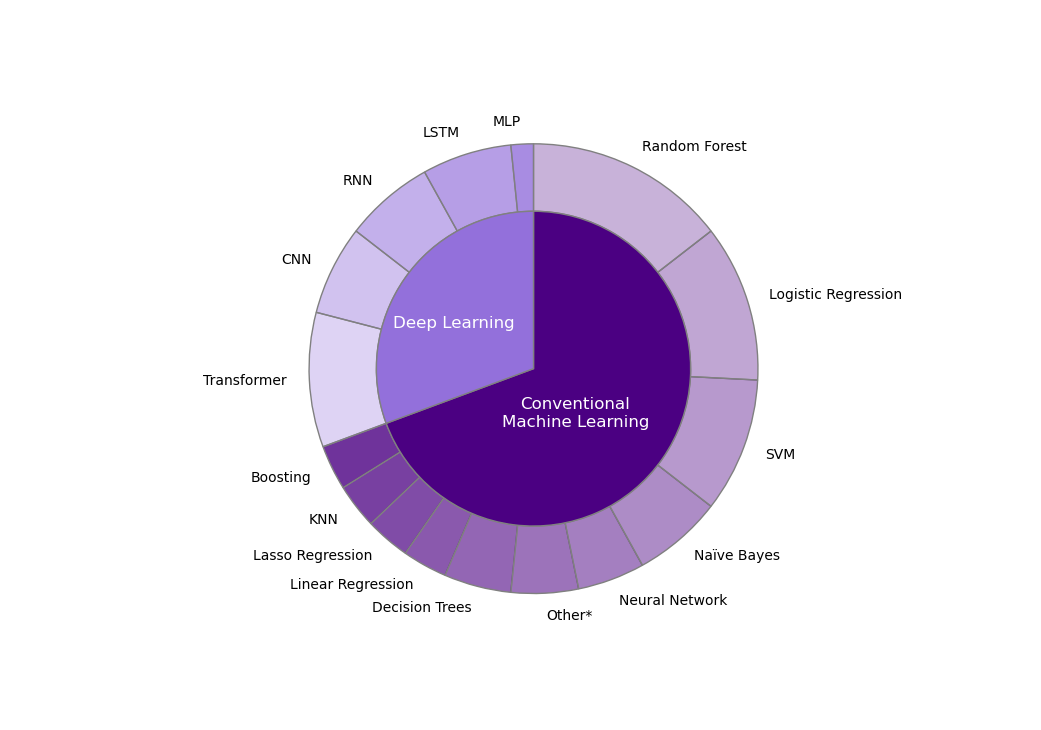


*Other includes latent Dirichlet allocation, ridge regression, and stepwise regression.

Figure S12. Relative Proportions of Machine Learning NLP Algorithms Used for Phenotyping/Severity


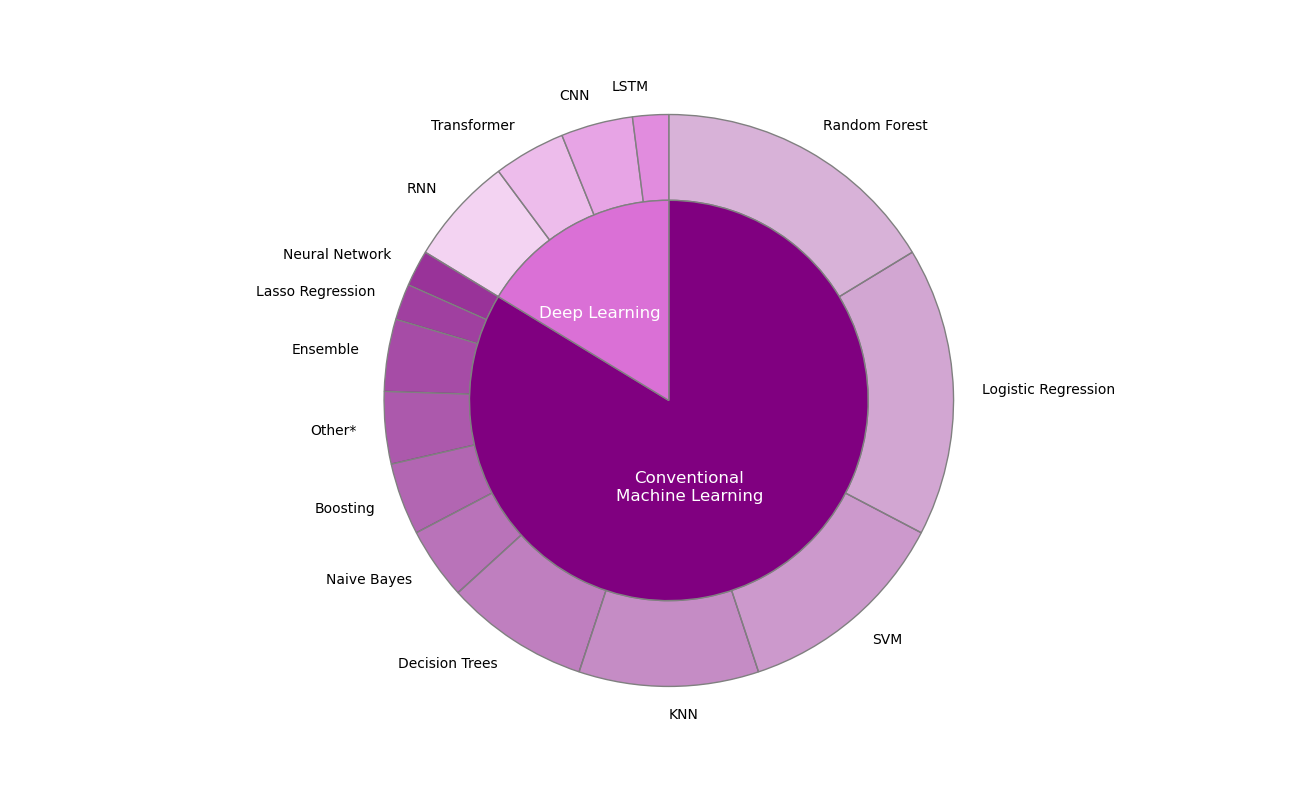


*Other includes unspecified neural network with unspecified number of layers and stepwise regression.

Figure S13. Relative Proportions of Machine Learning NLP Algorithms Used for Prognosis/Risk Stratification


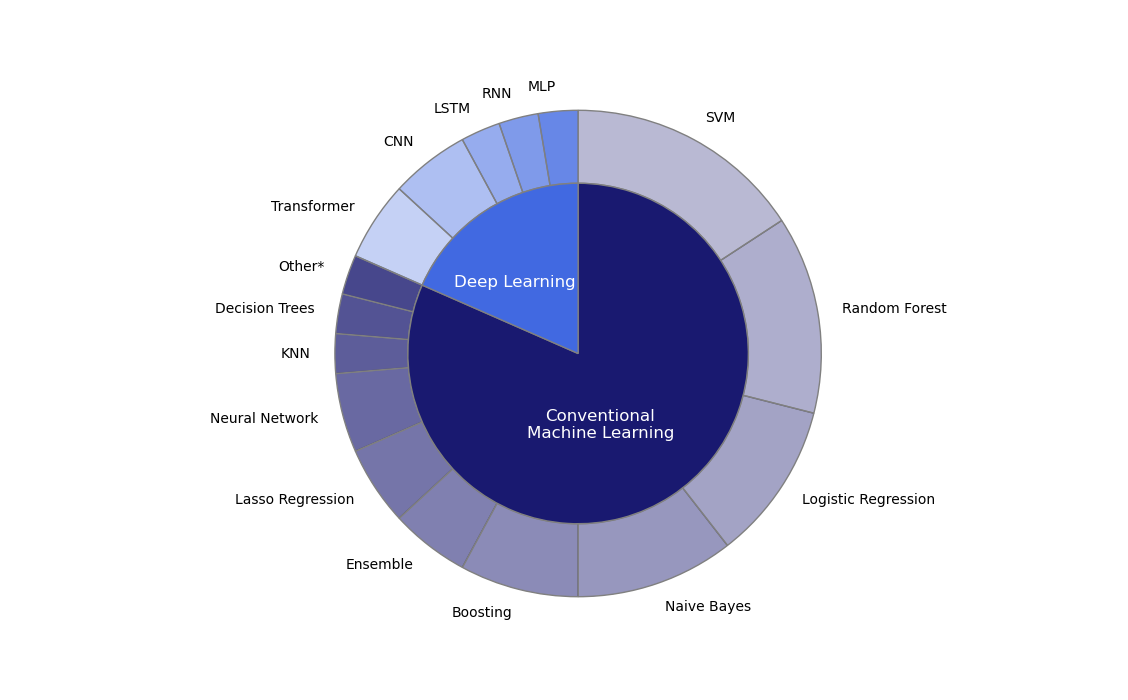


*Other includes unspecified penalized logistic regression method.

Figure S14. Relative Proportions of Machine Learning NLP Algorithms Used for Treatment


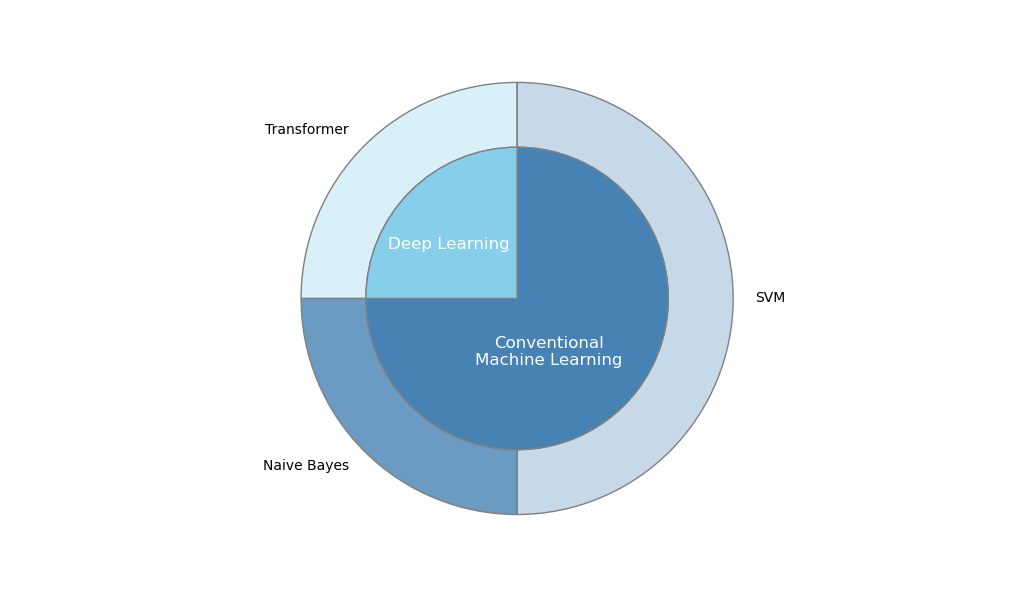


Performance of Externally Validated Studies

| **Author** | **Condition** | **Type of Model** | **Outcome** | **Measure of Performance** | **Performance** |
| --- | --- | --- | --- | --- | --- |
| Miller et al [19] | Stroke | BERT (best performing) | Radiographic complications of ischemic stroke | AUPRC | **External Validation Set 1:**  Edema, 0.99 (0.99, 1.00); Midline shift, 1.00 (1.00, 1.00); Hemorrhagic conversion, 0.89 (0.85, 0.93); Parenchymal hematoma, 0.78 (0.75, 0.82); Intraventricular hemorrhage, 0.59 (0.23, 0.96)  **External Validation Set 2:**  Edema, 0.94 (0.91, 0.97); Midline shift, .98 (0.95, 1.00); Hemorrhagic conversion, 0.90 (0.86, 0.93); Parenchymal hematoma, 0.76 (0.69, 0.84); Intraventricular hemorrhage, 0.89 (0.67, 1.00) |
| Li et al [16] | Stroke | Random forest | Acute or subacute ischemic stroke cases before and during COVID-19 | Accuracy, F1 | Accuracy, 0.95 (0.94-0.96); F1, 0.66 (0.59-0.72) |
| Zhao et al [21] | Stroke | Random forest (best performing) | Incidence of stroke, stroke subtypes | PPV, NPV | PPV, 0.86 (0.74-0.93); NPV without ICD codes, 1.00 (0.92-1.00); NPV with ICD codes, 0.92 (0.90-0.98) |
| Barbour et al [17] | Epilepsy | Rule-based | Risk factors for SUDEP | F1, F1 with sensitivity analysis (removing three boilerplate phrases) | F1 range, 0.51-0.81; F1 range with sensitivity analysis, 0.79-0.89 |
| Deng et al [18] | Stroke | BERT | Performance of system to generate ICH treatment plan | Accuracy, AUC-ROC | Accuracy, 88.55% (88.15-88.94); AUC-ROC, 0.887 (0.884-0.891) |
| Sung et al [20] | Stroke | BERT + Random forest | Prediction of poor functional outcome after acute ischemic stroke | AUC-ROC | Using HPI, 0.792; Using CT reports, 0.658; Using HPI and CT reports, 0.798 |
| Ong et al [22] | Stroke | RNN | Ischemic stroke presence, location, and acuity | AUC-ROC | Stroke presence, 0.92 (0.908-0.932); Location, 0.89 (0.99-0.905); Acuity, 0.93 (0.906-0.946) |

Sources of Funding

| **Non-sponsored** | n=4, 9.8% |
| --- | --- |
| **Not reported** | n=4, 9.8% |
| **Private only** | n=9, 22.0% |
| **Public only** | n=12, 29.3% |
| **Public and private** | n=12, 29.3% |
